# Supplementary material for: Immunity Awareness—Strategies to Improve the Degree of Acceptance of Vaccines: A Systematic Review
Source: Vaccines (Basel). 2025 Jun 7;13(6):618. doi: 10.3390/vaccines13060618 (PMC12197436; doi:10.3390/vaccines13060618)
Supplement: Supplementary file 1 [file vaccines-13-00618-s001.zip › vaccines-3647861-supplementary.pdf]

vaccines

Systematic Review

Immunity Awareness—Strategies to Improve the Degree of Acceptance of Vaccines: A Systematic Review

Alejandro Martínez-Serrano [1,2], Montserrat Pulido-Fuentes [3,4,*], Blanca Notario-Pacheco [1,4,5], Ana María Palmar-Santos [6], Ana Isabel Cobo-Cuenca [4,7] and Ana Díez-Fernández [1,4,5]

1 Social and Health Care Research Center, Universidad de Castilla-La Mancha, 16071 Cuenca, Spain
2 Servicio de Salud de Castilla-La Mancha, 02008 Albacete, Spain
3 Facultad de Ciencias de la Salud, Universidad de Castilla-La Mancha, 45600 Talavera de la Reina, Spain
4 Department of Nursing, Physiotherapy and Occupational Therapy, Universidad de Castilla-La Mancha, 16002 Cuenca, Spain
5 Facultad de Enfermería de Cuenca, Universidad de Castilla-La Mancha, 16002 Cuenca, Spain
6 Department of Nursing, Universidad Autónoma de Madrid, 28029 Madrid, Spain; ana.palmar@uam.es
7 Group IMCU, Faculty of Physiotherapy and Nursing, Universidad de Castilla-La Mancha, 45071 Toledo, Spain
* Correspondence: montserrat.pulido@uclm.es

Table S1. PRISMA 2020 Checklist

Section and Topic	Item #	Checklist item	Location where item is reported
**TITLE**			
Title	1	Identify the report as a systematic review.	1
**ABSTRACT**			
Abstract	2	See the PRISMA 2020 for Abstracts checklist.	Table S2
**INTRODUCTION**			
Rationale	3	Describe the rationale for the review in the context of existing knowledge.	1-2
Objectives	4	Provide an explicit statement of the objective(s) or question(s) the review addresses.	2
**METHODS**			
Eligibility criteria	5	Specify the inclusion and exclusion criteria for the review and how studies were grouped for the syntheses.	3
Information sources	6	Specify all databases, registers, websites, organisations, reference lists and other sources searched or consulted to identify studies. Specify the date when each source was last searched or consulted.	3
Search strategy	7	Present the full search strategies for all databases, registers and websites, including any filters and limits used.	3
Selection process	8	Specify the methods used to decide whether a study met the inclusion criteria of the review, including how many reviewers screened each record and each report retrieved, whether they worked independently, and if applicable, details of automation tools used in the process.	3
Data collection process	9	Specify the methods used to collect data from reports, including how many reviewers collected data from each report, whether they worked independently, any processes for obtaining or confirming data from study investigators, and if applicable, details of automation tools used in the process.	4
Data items	10a	List and define all outcomes for which data were sought. Specify whether all results that were compatible with each outcome domain in each study were sought (e.g. for all measures, time points, analyses), and if not, the methods used to decide which results to collect.	3
	10b	List and define all other variables for which data were sought (e.g. participant and intervention characteristics, funding sources). Describe any assumptions made about any missing or unclear information.	3
Study risk of bias assessment	11	Specify the methods used to assess risk of bias in the included studies, including details of the tool(s) used, how many reviewers assessed each study and whether they worked independently, and if applicable, details of automation tools used in the process.	5
Effect measures	12	Specify for each outcome the effect measure(s) (e.g. risk ratio, mean difference) used in the synthesis or presentation of results.	4
Synthesis methods	13a	Describe the processes used to decide which studies were eligible for each synthesis (e.g. tabulating the study intervention characteristics and comparing against the planned groups for each synthesis (item #5)).	3-4
	13b	Describe any methods required to prepare the data for presentation or synthesis, such as handling of missing summary statistics, or data conversions.	4
	13c	Describe any methods used to tabulate or visually display results of individual studies and syntheses.	4
	13d	Describe any methods used to synthesize results and provide a rationale for the choice(s). If meta-analysis was performed, describe the model(s), method(s) to identify the presence and extent of statistical heterogeneity, and software package(s) used.	4
	13e	Describe any methods used to explore possible causes of heterogeneity among study results (e.g. subgroup analysis, meta-regression).	-
	13f	Describe any sensitivity analyses conducted to assess robustness of the synthesized results.	4-5

Section and Topic	Item #	Checklist item	Location where item is reported
Reporting bias assessment	14	Describe any methods used to assess risk of bias due to missing results in a synthesis (arising from reporting biases).	5
Certainty assessment	15	Describe any methods used to assess certainty (or confidence) in the body of evidence for an outcome.	5
**RESULTS**			
Study selection	16a	Describe the results of the search and selection process, from the number of records identified in the search to the number of studies included in the review, ideally using a flow diagram.	5-6
	16b	Cite studies that might appear to meet the inclusion criteria, but which were excluded, and explain why they were excluded.	5
Study characteristics	17	Cite each included study and present its characteristics.	Table S5
Risk of bias in studies	18	Present assessments of risk of bias for each included study.	Table S7-S9
Results of individual studies	19	For all outcomes, present, for each study: (a) summary statistics for each group (where appropriate) and (b) an effect estimate and its precision (e.g. confidence/credible interval), ideally using structured tables or plots.	Table S7-S9
Results of syntheses	20a	For each synthesis, briefly summarise the characteristics and risk of bias among contributing studies.	5-8
	20b	Present results of all statistical syntheses conducted. If meta-analysis was done, present for each the summary estimate and its precision (e.g. confidence/credible interval) and measures of statistical heterogeneity. If comparing groups, describe the direction of the effect.	-
	20c	Present results of all investigations of possible causes of heterogeneity among study results.	-
	20d	Present results of all sensitivity analyses conducted to assess the robustness of the synthesized results.	8
Reporting biases	21	Present assessments of risk of bias due to missing results (arising from reporting biases) for each synthesis assessed.	8
Certainty of evidence	22	Present assessments of certainty (or confidence) in the body of evidence for each outcome assessed.	Table S7-S9
**DISCUSSION**			
Discussion	23a	Provide a general interpretation of the results in the context of other evidence.	8-9
	23b	Discuss any limitations of the evidence included in the review.	11
	23c	Discuss any limitations of the review processes used.	11
	23d	Discuss implications of the results for practice, policy, and future research.	10-11
**OTHER INFORMATION**			
Registration and protocol	24a	Provide registration information for the review, including register name and registration number, or state that the review was not registered.	2
	24b	Indicate where the review protocol can be accessed, or state that a protocol was not prepared.	2
	24c	Describe and explain any amendments to information provided at registration or in the protocol.	2
Support	25	Describe sources of financial or non-financial support for the review, and the role of the funders or sponsors in the review.	12
Competing interests	26	Declare any competing interests of review authors.	12
Availability of data, code and other materials	27	Report which of the following are publicly available and where they can be found: template data collection forms; data extracted from included studies; data used for all analyses; analytic code; any other materials used in the review.	2

Table S2. PRISMA 2020 for Abstracts Checklist

Section and Topic	Item #	Checklist item	Reported (Yes/No)
**TITLE**			
Title	1	Identify the report as a systematic review.	Yes
**BACKGROUND**			
Objectives	2	Provide an explicit statement of the main objective(s) or question(s) the review addresses.	Yes
**METHODS**			
Eligibility criteria	3	Specify the inclusion and exclusion criteria for the review.	Yes
Information sources	4	Specify the information sources (e.g. databases, registers) used to identify studies and the date when each was last searched.	Yes
Risk of bias	5	Specify the methods used to assess risk of bias in the included studies.	No
Synthesis of results	6	Specify the methods used to present and synthesise results.	Yes
**RESULTS**			
Included studies	7	Give the total number of included studies and participants and summarise relevant characteristics of studies.	Yes
Synthesis of results	8	Present results for main outcomes, preferably indicating the number of included studies and participants for each. If meta-analysis was done, report the summary estimate and confidence/credible interval. If comparing groups, indicate the direction of the effect (i.e. which group is favoured).	Yes
**DISCUSSION**			
Limitations of evidence	9	Provide a brief summary of the limitations of the evidence included in the review (e.g. study risk of bias, inconsistency and imprecision).	No
Interpretation	10	Provide a general interpretation of the results and important implications.	Yes
**OTHER**			
Funding	11	Specify the primary source of funding for the review.	No
Registration	12	Provide the register name and registration number.	No

Table S3. Search strategy

Database	Search
MEDLINE	"vaccine hesitancy" AND education AND strategies
	vaccination AND refusal AND education AND strategies
Dialnet	vacuna AND rechazo AND estrategias
	vacuna AND rechazo AND educación
	anti AND vacuna AND educación
Scielo	vacuna AND rechazo
	vaccine AND refusal AND strategies
CINAHL	vaccine hesitancy AND strategies AND education
	vaccination refusal AND strategies AND education
CENTRAL	vaccine hesitancy AND education
	vaccine AND education AND strategies

Table S4. List of excluded studies

Number	Reference	Reason of exclusion
1	Abdel-Qader DH, Al Meslamani AZ, Al Mazrouei N, El-Shara AA, El Sharu H, Merghani Ali E, et al. Virtual coaching delivered by pharmacists to prevent COVID-19 transmission. Hosp Pharm 2022;57:300-8	Article that develops interventions aimed at healthcare staff to improve communication with hesitant patients.
2	Abdel-Qader DH, Hayajneh W, Albassam A, Obeidat NM, Belbeisi AM, Al Mazrouei N, et al. Pharmacists-physicians collaborative intervention to reduce vaccine hesitancy and resistance: A randomized controlled trial. Vaccine X 2022;10:100135	COVID-19 vaccine intervention.
3	Aborode AT, Fajemisin EA, Ekwebelem OC, Tsagkaris C, Taiwo EA, Uwishema O, et al. Vaccine hesitancy in Africa: causes and strategies to the rescue. Ther Adv Vaccines Immunother 2021;9:25151355211047510	Analysis article of effective education methods.
4	Afreh OK, Angwaawie P, Attivor E, Boateng LA, Brackstone K, Head MG, et al. Examining confidence and hesitancy towards COVID-19 vaccines: A cross-sectional survey using in-person data collection in rural Ghana. Vaccine 2023;41:2113-9	Cross-sectional study aimed to understand the factors that contribute to hesitancy.
5	Aggarwal M, Kokorelias KM, Glazier RH, Katz A, Shiers-Hanley JE, Upshur REG. What is the role of primary care in the COVID-19 vaccine roll-out and the barriers and facilitators to an equitable vaccine roll-out? A rapid scoping review of nine jurisdictions. BMJ Open 2023;13:e065306	Systematic review.
6	Ahillan T, Emmerson M, Swift B, Golamgouse H, Song K, Roxas A, et al. COVID-19 in the homeless population: a scoping review and meta-analysis examining differences in prevalence, presentation, vaccine hesitancy and government response in the first year of the pandemic. BMC Infect Dis 2023;23:155	Systematic review.
7	Aiyer I, Shaik L, Kashyap R, Surani S. COVID-19 misinformation: A potent co-factor in the COVID-19 pandemic. Cureus 2022;14:e30026	Analysis article of effective education methods.
8	Alcendor DJ, Matthews-Juarez P, Smoot D, Hildreth JEK, Tabatabai M, Wilus D, et al. The COVID-19 vaccine and pregnant minority women in the US: Implications for improving vaccine confidence and uptake. Vaccines (Basel) 2022;10:2122	Analysis article of effective education methods.
9	Alharbi M, Alharbi NM, Almutairi SM, Alharbi MK, Alsaud JS, Alnssyan B, et al. Acceptance towards COVID-19 vaccine among Qassim populations: A cross-sectional study. J Family Med Prim Care 2023;12:213-22	Cross-sectional study aimed to understand the factors that contribute to hesitancy.
10	Almojaibel A, Ansari K, Alzahrani Y, Alquaimi M, Farooqi F, Alqurashi Y. COVID-19 vaccine hesitancy in the Saudi Arabian population. J Med Life 2023;16:101-9	Cross-sectional study aimed to understand the factors that contribute to hesitancy.
11	Aloweidi A, Bsisu I, Suleiman A, Abu-Halaweh S, Almustafa M, Aqel M, et al. Hesitancy towards COVID-19 vaccines: An analytical cross-sectional study. Int J Environ Res Public Health 2021;18:5111	Analysis article of effective education methods.
12	AlShurman BA, Khan AF, Mac C, Majeed M, Butt ZA. What demographic, social, and contextual factors influence the intention to use COVID-19 vaccines: A scoping review. Int J Environ Res Public Health 2021;18:9342	Systematic review.
13	Amani A, Atuhebwe P, Mboussou FF, Ngoy N, M'boufoungou NE, Osei-Sarpong F, et al. A rapid increase in coverage of COVID-19 vaccination, Central African Republic. Bull World Health Organ 2023;101:431-6	Article without methods to measure the level of effectiveness.
14	Anakpo G, Mishi S. Hesitancy of COVID-19 vaccines: Rapid systematic review of the measurement, predictors, and preventive strategies. Hum Vaccin Immunother 2022;18:2074716	Systematic review.
15	Arghittu A, Dettori M, Dempsey E, Deiana G, Angelini C, Bechini A, et al. Health communication in COVID-19 era: Experiences from the Italian VaccinarSì Network websites. Int J Environ Res Public Health 2021;18:5642	Analysis article of effective education methods.

Number	Reference	Reason of exclusion
16	Arnett MC, Evans, Stull C. Dental Hygiene Students' Perceptions Regarding the Importance of and Confidence with Using Brief Motivational Interviewing during HPV Patient Counseling. Journal of dental hygiene : JDH 2022;96	Article that develops interventions aimed at healthcare staff to improve communication with hesitant patients.
17	Ata Teneler A, Ayhan F, Zaim E, Ozek U. Un estudio comunitario en el distrito central de Giresun: dudas sobre la vacuna COVID-19. Hum Vaccin Immunother 2022;18:2092362	Cross-sectional study aimed to understand the factors that contribute to hesitancy.
18	AuYoung M, Rodriguez Espinosa P, Chen W-T, Juturu P, Young M-EDT, Casillas A, et al. Addressing racial/ethnic inequities in vaccine hesitancy and uptake: lessons learned from the California alliance against COVID-19. J Behav Med 2023;46:153-66	Analysis article of effective education methods.
19	Azzari C, Diez-Domingo J, Eisenstein E, Faust SN, Konstantopoulos A, Marshall GS, et al. Experts' opinion for improving global adolescent vaccination rates: a call to action. Eur J Pediatr 2020;179:547-53	Opinion-based article.
20	Bacong AM, Horse AJY, Lee E, Đoàn LN, Saw A. Modes of COVID-19 information and vaccine hesitancy among Asian Americans: The moderating role of exposure to cyberbullying. AJPM Focus 2023;2:100130	Cross-sectional study aimed to understand the factors that contribute to hesitancy.
21	Badua AR, Caraquel KJ, Cruz M, Narvaez RA. Vaccine literacy: A concept analysis. Int J Ment Health Nurs 2022;31:857-67	Analysis article of effective education methods.
22	Barnawi NA, Alraqei B, Hilwan A, Al-Otibi M, Alsubaie R, Altowymy S, et al. Impact of a video-based educational intervention on the levels of knowledge and concerns about COVID-19 vaccination. Vaccines (Basel) 2023;11:727	COVID-19 vaccine intervention.
23	Barton SM, Calhoun AW, Bohnert CA, Multerer SM, Statler VA, Bryant KA, et al. Standardized Vaccine-Hesitant Patients in the Assessment of the Effectiveness of Vaccine Communication Training. The Journal of pediatrics 2022;241	Article that develops interventions aimed at healthcare staff to improve communication with hesitant patients.
24	Bazzi AR, Harvey-Vera A, Buesig-Stamos T, Abramovitz D, Vera CF, Artamonova I, et al. Study protocol for a pilot randomized controlled trial to increase COVID-19 testing and vaccination among people who inject drugs in San Diego County. Addict Sci Clin Pract 2022;17:48	Intervention protocol.
25	Berry SD, Goldfeld KS, McConeghy K, Gifford D, Davidson HE, Han L, et al. Evaluating the findings of the IMPACT-C randomized clinical trial to improve COVID-19 vaccine coverage in skilled nursing facilities. JAMA Intern Med 2022;182:324-31	COVID-19 vaccine intervention.
26	Bester JC. Not a matter of parental choice but of social justice obligation: Children are owed measles vaccination: XXXX. Bioethics 2018;32:611-9	Opinion-based article.
27	Bocquier A, Michel M, Giraudeau B, Bonnay S, Gagneux-Brunon A, Gauchet A, et al. Impact of a school-based and primary care-based multicomponent intervention on HPV vaccination coverage among French adolescents: a cluster randomised controlled trial protocol (the PrevHPV study). BMJ Open 2022;12:e057943	Intervention protocol.
28	Bradley-Ewing A, Lee BR, Doctor JN, Meredith G, Goggin K, Myers A. A pilot intervention combining assessment and feedback with communication training and behavioral nudges to increase HPV vaccine uptake. Hum Vaccin Immunother 2022;18:1885968	Analysis article of effective education methods.
29	Brunelli L, Antinolfi F, Malacarne F, Cocconi R, Brusaferro S. A wide range of strategies to cope with healthcare workers' vaccine hesitancy in A North-eastern Italian Region: Are they enough? Healthcare (Basel) 2020;9:4	Analysis article of effective education methods.
30	Butow P, Shaw J, Bartley N, Milch V, Sathiaraj R, Turnbull S, et al. Vaccine hesitancy in cancer patients: A rapid review. Patient Educ Couns 2023;111:107680	Systematic review.
31	Caballero A, Leath KJ, Staton AD. Strategic combination of theory, plain language, and trusted messengers contribute to COVID-19 vaccine uptake: Lessons learned from development and dissemination of a community toolkit. Vaccines (Basel) 2023;11:1064	Article that develops interventions aimed at healthcare staff to improve communication with hesitant patients.
32	Cadeddu C, Sapienza M, Castagna C, Regazzi L, Paladini A, Ricciardi W, et al. Vaccine hesitancy and trust in the scientific community in Italy: Comparative analysis from two recent surveys. Vaccines (Basel) 2021;9:1206	Cross-sectional study aimed to understand the factors that contribute to hesitancy.
33	Carney PA, Hatch B, Stock I, Dickinson C, Davis M, Larsen R, et al. A stepped-wedge cluster randomized trial designed to improve completion of HPV vaccine series and reduce missed opportunities to vaccinate in rural primary care practices. Implement Sci 2019;14:30	Intervention protocol.
34	Casey SM, Burrowes SAB, Hall T, Dobbins S, Ma M, Bano R, et al. Healthcare workers' attitudes on mandates, incentives, and strategies to improve COVID-19 vaccine uptake: A mixed methods study. Hum Vaccin Immunother 2022;18:2144048	Cross-sectional study aimed to understand the factors that contribute to hesitancy.
35	Cataldi JR, O'Leary ST. Addressing vaccine concerns: A hopeful path forward for vaccine confidence. Am J Public Health 2021;111:556-8	Analysis article of effective education methods.

Number	Reference	Reason of exclusion
36	Chau JPC, Lo SHS, Choi KC, Lee VWY, Lui GCY, Chan KM, et al. Effects of a multidisciplinary team-led school-based human papillomavirus vaccination health-promotion programme on improving vaccine acceptance and uptake among female adolescents: A cluster randomized controlled trial: A cluster randomized controlled trial. Medicine (Baltimore) 2020;99:e22072	Intervention protocol.
37	Chen S, Forster S, Yang J, Yu F, Jiao L, Gates J, et al. Animated, video entertainment-education to improve vaccine confidence globally during the COVID-19 pandemic: an online randomized controlled experiment with 24,000 participants. Trials 2022;23:161	Intervention protocol.
38	Chin J, Zhou Y, Chen CL, Lomiguen CM, McClelland S, Lee-Wong M. Influenza vaccination quality improvement as a model for COVID-19 prophylaxis. Cureus 2021;13:e12549	Cross-sectional study aimed to understand the factors that contribute to hesitancy.
39	Corley AMS, Gomes SM, Martin KJ, Watkins S, Lindsey K, Frenck RW Jr, et al. Evaluation of a community COVID-19 vaccine ambassador train-the-trainer program. J Immigr Minor Health 2023; 5:1-5.	Article that develops interventions aimed at healthcare staff to improve communication with hesitant patients.
40	Cunningham-Erves J, Wilkins CH, Dempsey AF, Jones JL, Thompson C, Edwards K, et al. Development of a tailored mobile phone-based intervention to facilitate parent-child communication and build human Papillomavirus vaccine confidence: Formative qualitative study. JMIR Form Res 2023;7:e43041	Intervention protocol.
41	Davies C, Stoney T, Hutton H, Parrella A, Kang M, Macartney K, et al. School-based HPV vaccination positively impacts parents' attitudes toward adolescent vaccination. Vaccine 2021;39:4190-8	Article without methods to measure the level of effectiveness.
42	Decker F. Communicating with COVID-Vaccine: Hesitant patients. Family Doctor: A Journal of the New York State Academy of Family Physicians 2021///Summer2021;10:11-4	Analysis article of effective education methods.
43	Demeke J, Ramos SR, McFadden SM, Dada D, Nguemo Djiometio J, Vlahov D, et al. Strategies that promote equity in COVID-19 vaccine uptake for Latinx communities: A review. J Racial Ethn Health Disparities 2023;10:1349-57	Systematic review.
44	Dempsey AF, Wagner N, Narwaney K, Pyrzanowski J, Kwan BM, Kraus C, et al. «Reducing Delays In Vaccination» (REDIVAC) trial: a protocol for a randomised controlled trial of a web-based, individually tailored, educational intervention to improve timeliness of infant vaccination. BMJ Open 2019;9:e027968	Intervention protocol.
45	Desmond A, Offit PA. The antivax movement and what allergists can do. Ann Allergy Asthma Immunol 2020;125:8-9.e2	Analysis article of effective education methods.
46	Domgaard S, Park M. Combating misinformation: The effects of infographics in verifying false vaccine news. Health Educ J 2021;80:974-86	Analysis article of effective education methods.
47	Dominguez ME, Macias-Carlos D, Montoya JA, Plant A, Neffa-Creech D. Integrated multicultural media campaign to increase COVID-19 education and vaccination among Californians, 2021. Am J Public Health 2022;112:1389-93	COVID-19 vaccine intervention.
48	Dotlic J, Jeremic Stojkovic V, Cummins P, Milic M, Gazibara T. Enhancing COVID-19 vaccination coverage using financial incentives: arguments to help health providers counterbalance erroneous claims. Epidemiol Health 2021;43:e2021081	Opinion-based article.
49	Duvall A. Improving Influenza Vaccination Rates Among Pediatric Hematology and Oncology Inpatients. Pediatr Nurs 2019;45:142	Article that develops interventions aimed at healthcare staff to improve communication with hesitant patients.
50	Ekezie W, Connor A, Gibson E, Khunti K, Kamal A. A systematic review of behaviour change techniques within interventions to increase vaccine uptake among ethnic minority populations. Vaccines (Basel) 2023;11	Systematic review.
51	Erwin PC, Linares-Pérez N, Verez-Bencomo V. Vaccination achievements of Cuba versus the United States exposed by the 2019 measles epidemic. Am J Public Health 2020;110:467-9	Opinion-based article.
52	Feemster KA, Head KJ, Panozzo CA, O'Dell SM, Zimet GD, Kornides ML. Efficacy of tailored messages to improve behavioral intent to accept HPV vaccination among mothers may be moderated by sociodemographics. Prev Med Rep 2021;23:101413	Article without methods to measure the level of effectiveness.
53	Finney Rutten LJ, Radecki Breitkopf C, St Sauver JL, Croghan IT, Jacobson DJ, Wilson PM, et al. Evaluating the impact of multilevel evidence-based implementation strategies to enhance provider recommendation on human papillomavirus vaccination rates among an empaneled primary care patient population: a study protocol for a stepped-wedge cluster randomized trial. Implement Sci 2018;13:96	Intervention protocol.
54	Fisher WA, Gilca V, Murti M, Orth A, Garfield H, Roumeliotis P, et al. Continuing medical education improves physician communication skills and increases likelihood of pediatric vaccination: Findings from the pediatric influenza vaccination optimization trial (PIVOT)-II. Vaccines (Basel) 2022;11:17	Article that develops interventions aimed at healthcare staff to improve communication with hesitant patients.
55	Gagneur A. Motivational interviewing: A powerful tool to address vaccine hesitancy. Can Commun Dis Rep 2020;46:93-7	Analysis article of effective education methods.

Number	Reference	Reason of exclusion
56	Gagneur A, Quach C, Boucher FD, Tapiero B, De Wals P, Farrands A, et al. Promoting vaccination in the province of Québec: the PromoVaQ randomized controlled trial protocol. BMC Public Health 2019;19	Intervention protocol.
57	Garcia-Grossman IR, Gransee L, Williams B. Strategies for addressing vaccine hesitancy within California state prisons in 2021 and beyond. Am J Public Health 2022;112:1543-5	COVID-19 vaccine intervention.
58	Gillard CJ, Al-Dahir S, Earls M, Singleton B. A culturally competent vaccine hesitancy educational model for community pharmacists to increase vaccine uptake, Louisiana, 2021-2022. Am J Public Health 2022;112:S900-3	Article that develops interventions aimed at healthcare staff to improve communication with hesitant patients.
59	Gobbo ELS, Hanson C, Abunnaja KSS, van Wees SH. Do peer-based education interventions effectively improve vaccination acceptance? a systematic review. BMC Public Health 2023;23:1354	Systematic review.
60	Goldman RD, Staubli G, Cotanda CP, Brown JC, Hoeffe J, Seiler M, et al. Factors associated with parents' willingness to enroll their children in trials for COVID-19 vaccination. Hum Vaccin Immunother 2021;17:1607-11	Cross-sectional study aimed to understand the factors that contribute to hesitancy.
61	Haase N, Schmid P, Betsch C. Impact of disease risk on the narrative bias in vaccination risk perceptions. Psychol Health 2020;35:346-65	Analysis article of effective education methods.
62	Habersaat KB, Jackson C. Understanding vaccine acceptance and demand-and ways to increase them. Bundesgesundheitsblatt Gesundheitsforschung Gesundheitsschutz 2020;63:32-9	Analysis article of effective education methods.
63	Hadjipanayis A, Dornbusch HJ, Grossman Z, Theophilou L, Brierley J. Mandatory vaccination: a joint statement of the Ethics and Vaccination working groups of the European Academy of Paediatrics. Eur J Pediatr 2020;179:683-7	Opinion-based article.
64	Herzog NK, Vasireddy H, Drenner DA, Rose JP. The effects of social-media based social comparison information and similarity mindsets on COVID-19 vaccination uptake cognitions. J Behav Med 2023;46:276-89	COVID-19 vaccine intervention.
65	Homer CSE, Javid N, Wilton K, Bradfield Z. Vaccination in pregnancy: The role of the midwife. Front Glob Womens Health 2022;3:929173	Analysis article of effective education methods.
66	Huang Y, Green MC. Reducing COVID-19 vaccine hesitancy among African Americans: the effects of narratives, character's self-persuasion, and trust in science. J Behav Med 2023;46:290-302	COVID-19 vaccine intervention.
67	Hunt I de V, Dunn T, Mahoney M, Chen M, Nava V, Linos E. A social media–based Public Health campaign encouraging COVID-19 vaccination across the United States. Am J Public Health 2022;112:1253-6	Article without methods to measure the level of effectiveness.
68	Janitz AE, Neil JM, Bray LA, Jervis LL, Ross L, Campbell JE, et al. CATCH-UP vaccines: protocol for a randomized controlled trial using the multiphase optimization strategy (MOST) framework to evaluate education interventions to increase COVID-19 vaccine uptake in Oklahoma. BMC Public Health 2023;23:1146	Intervention protocol.
69	Johnson SA, Spates JD, Smith UD, Harris CM, Simmons M, Domingue A, et al. COVID-19 Vaccine Hesitancy among People of Color: Meeting the Needs in Florida. ABNFF Journal 2023;2:15-20	Systematic review.
70	Kaufman J, Ryan R, Lewin S, Bosch-Capblanch X, Glenton C, Cliff J, et al. Identification of preliminary core outcome domains for communication about childhood vaccination: An online Delphi survey. Vaccine 2018;36:6520-8	Analysis article of effective education methods.
71	Kaufman J, Ryan R, Walsh L, Horey D, Leask J, Robinson P, et al. Face-to-face interventions for informing or educating parents about early childhood vaccination. Cochrane Database Syst Rev 2018;5:CD010038	Systematic review.
72	Kiyota E. A Global Perspective. Generations 2022;46:1-15	Analysis article of effective education methods.
73	Kornides, M. L., Badlis, S., Head, K. J., Putt, M., Cappella, J., & Gonzalez-Hernadez, G. (2023). Exploring content of misinformation about HPV vaccine on twitter. Journal of Behavioral Medicine, 46(1-2), 239–252	Cross-sectional study aimed to understand the factors that contribute to hesitancy.
74	Kregar Velikonja N, Dobrowolska B, Stanisavljević S, Erjavec K, Globevnik Velikonja V, Verdenik I. Attitudes of nursing students towards vaccination and other preventive measures for limitation of COVID-19 pandemic: Cross-sectional study in three European countries. Healthcare (Basel) 2021;9:781	Cross-sectional study aimed to understand the factors that contribute to hesitancy.
75	Kwan BM, Pyrzanowski J, Sevick C, Wagner NM, Resnicow K, Glanz JM, et al. Exploring mechanisms of a web-based values-tailored childhood vaccine promotion intervention trial: Effects on parental vaccination values, attitudes, and intentions. Appl Psychol Health Well Being 2022;14:158-75	Analysis article of effective education methods.
76	Larson HJ, Broniatowski DA. Why debunking misinformation is not enough to change people's minds about vaccines. Am J Public Health 2021;111:1058-60	Opinion-based article.
77	Li L, Wood CE, Kostkova P. Vaccine hesitancy and behavior change theory-based social media interventions: a systematic review. Transl Behav Med 2022;12:243-72	Systematic review.
78	Li S, Xia Y, Zhao W, Miao X, Xu Q. Self-affirmation increases acceptance of information on COVID-19 vaccines and promotes vaccination intention. J Behav Med 2023;46:303-10	COVID-19 vaccine intervention.

Number	Reference	Reason of exclusion
79	Lip A, Pateman M, Fullerton MM, Chen HM, Bailey L, Houle S, et al. Vaccine hesitancy educational tools for healthcare providers and trainees: A scoping review. Vaccine 2023;41:23-35	Systematic review.
80	Lockhart S, Dempsey AF, Pyrzanowski J, O'Leary ST, Barnard JG. Provider and parent perspectives on enhanced communication tools for human Papillomavirus vaccine-hesitant parents. Acad Pediatr 2018;18:776-82	Article that develops interventions aimed at healthcare staff to improve communication with hesitant patients.
81	MacDonald NE, Butler R, Dubé E. Addressing barriers to vaccine acceptance: an overview. Hum Vaccin Immunother 2018;14:218-24	Systematic review.
82	MacDonald NE, Dubé E. Addressing vaccine hesitancy in immunization programs, clinics and practices. Paediatr Child Health 2018;23:559-60	Analysis article of effective education methods.
83	MacDonald NE, Dubé E. Promoting immunization resiliency in the digital information age. Can Commun Dis Rep 2020;46:20-4	Analysis article of effective education methods.
84	Machado Júnior C, Mantovani DMN, de Sandes-Guimarães LV, Romeiro M do C, Furlaneto CJ, Bazanini R. Volatility of the COVID-19 vaccine hesitancy: sentiment analysis conducted in Brazil. Front Public Health 2023;11:1192155	Cross-sectional study aimed to understand the factors that contribute to hesitancy.
85	Madrigal JM, Johnson CA, Green J, Patel A. Using peer health educators to conduct community level surveillance of HPV vaccination status: Findings among women who live in medically underserved areas of Chicago. J Community Health 2020;45:1043-9	Cross-sectional study aimed to understand the factors that contribute to hesitancy.
86	Malik MN, Awan MS, Saleem T. Social mobilization campaign to tackle immunization hesitancy in Sargodha and Khushab districts of Pakistan. J Glob Health 2020;10:021302	Analysis article of effective education methods.
87	Malo TL, Hall ME, Brewer NT, Lathren CR, Gilkey MB. Why is announcement training more effective than conversation training for introducing HPV vaccination? A theory-based investigation. Implement Sci 2018;13:57	Article that develops interventions aimed at healthcare staff to improve communication with hesitant patients.
88	Mashinini DP, Lagerwey MD, Fogarty KJ, Potter RC. Methods of current practice: Qualitative analysis of intervention strategies utilized by vaccine waiver educators in Michigan. Policy Polit Nurs Pract 2022;23:249-58	Analysis article of effective education methods.
89	Mathur PK, Dolgin N. Crafting successful state-level public health interventions to vaccinate homeless populations during COVID-19. Am J Public Health 2022;112:601-3	Opinion-based article.
90	Merkley E, Loewen PJ. Assessment of communication strategies for mitigating COVID-19 vaccine-specific hesitancy in Canada. JAMA Netw Open 2021;4:e2126635	COVID-19 vaccine intervention.
91	Mondal P, Sinharoy A. The influence of pediatricians' recommendation on caregivers' COVID-19 vaccine acceptance for children: A nationwide cross-sectional survey study from USA. Front Pediatr 2023;11:1149125	Cross-sectional study aimed to understand the factors that contribute to hesitancy.
92	Nazari A, Hoseinnia M, Pirzadeh A, Salahshouri A. The correlation among COVID-19 vaccine acceptance, the ability to detect fake news, and e-health literacy. Health Lit Res Pract 2023;7:e130-8	Cross-sectional study aimed to understand the factors that contribute to hesitancy.
93	Nour R. A systematic review of methods to improve attitudes towards childhood vaccinations. Cureus 2019;11:e5067	Systematic review.
94	O'Leary ST, Spina CI, Spielvogle H, Robinson JD, Garrett K, Perreira C, et al. Development of PIVOT with MI: A motivational Interviewing-Based vaccine communication training for pediatric clinicians. Vaccine 2023;41:1760-7	Article that develops interventions aimed at healthcare staff to improve communication with hesitant patients.
95	Odone A, Gianfredi V, Sorbello S, Capraro M, Frascella B, Vigezzi GP, et al. The use of digital technologies to support vaccination programmes in Europe: State of the art and best practices from experts' interviews. Vaccines (Basel) 2021;9:1126	Analysis article of effective education methods.
96	Okuhara T, Ishikawa H, Okada M, Kato M, Kiuchi T. Japanese anti- versus pro-influenza vaccination websites: a text-mining analysis. Health Promot Int 2019;34:552-66	Analysis article of effective education methods.
97	Okuhara T, Okada H, Goto E, Tsunezumi A, Kagawa Y, Kiuchi T. Encouraging COVID-19 vaccination via an evolutionary theoretical approach: A randomized controlled study in Japan. Patient Educ Couns 2022;105:2248-55	COVID-19 vaccine intervention.
98	Okuhara T, Okada H, Goto E, Tsunezumi A, Kagawa Y, Kiuchi T. Encouraging HPV vaccination via an evolutionary theoretical approach: A randomized controlled study in Japan. Vaccines (Basel) 2022;10:701	Analysis article of effective education methods. Same database that Okuhara T, et al.
99	Omer SB, Benjamin RM, Brewer NT, Buttenheim AM, Callaghan T, Caplan A, et al. Promoting COVID-19 vaccine acceptance: recommendations from the Lancet Commission on Vaccine Refusal, Acceptance, and Demand in the USA. Lancet 2021;398:2186-92	Analysis article of effective education methods.
100	Opel DJ, Zhou C, Robinson JD, Henrikson N, Lepere K, Mangione-Smith R, et al. Impact of childhood vaccine discussion format over time on immunization status. Acad Pediatr 2018;18:430-6	Cross-sectional study aimed to understand the factors that contribute to hesitancy.

Number	Reference	Reason of exclusion
101	Orenstein EW, ElSayed-Ali O, Kandaswamy S, Masterson E, Blanco R, Shah P, et al. Evaluation of a clinical decision support strategy to increase seasonal influenza vaccination among hospitalized children before inpatient discharge. JAMA Netw Open 2021;4:e2117809	Article that develops interventions aimed at healthcare staff to improve communication with hesitant patients.
102	Ort A, Fahr A. Using efficacy cues in persuasive health communication is more effective than employing threats - An experimental study of a vaccination intervention against Ebola. Br J Health Psychol 2018;23:665-84	Article without methods to measure the level of effectiveness. Article not intended to improve confidence in vaccination.
103	Ouamba JP, Mbarga NF, Ciglenecki I, Ratnayake R, Tchiasso D, Finger F, et al. Implementation of targeted cholera response activities, Cameroon. Bull World Health Organ 2023;101:170-8	Article without methods to measure the level of effectiveness.
104	Oyo-Ita AE, Hanlon P, Nwankwo O, Bosch-Capblanch X, Arikpo D, Esu E, et al. Cost-effectiveness analysis of an intervention project engaging Traditional and Religious Leaders to improve uptake of childhood immunization in southern Nigeria. PLoS One 2021;16:e025727	Analysis article of effective education methods.
105	Pahud B, Elizabeth Williams S, Lee BR, Lewis KO, Middleton DB, Clark S, et al. A randomized controlled trial of an online immunization curriculum. Vaccine 2020;38:7299-30	Article that develops interventions aimed at healthcare staff to improve communication with hesitant patients.
106	Patel A, Resic A, Moore D. Improving Adult Immunization Rates Within Racial and Ethnic Minority Communities. fpm 2022;29:25-8	Analysis article of effective education methods.
107	Pihl GT, Ammentorp J, Johannessen H, Kjaergaard J, Nissen TN, Birk NM, et al. Mothers’ informational needs when deciding to have their newborn infant vaccinated with BCG. A Mixed-methods design. Scand J Caring Sci 2018;32:1118-26	Cross-sectional study aimed to understand the level of satisfaction with the information they had received.
108	Pires C. What is the state-of-the-art in clinical trials on vaccine hesitancy 2015-2020? Vaccines (Basel) 2021;9:348	Systematic review.
109	Pirrotta L, Guidotti E, Tramontani C, Bignardelli E, Venturi G, De Rosis S. COVID-19 vaccinations: An overview of the Italian national health system’s online communication from a citizen perspective. Health Policy 2022;126:970-9	Cross-sectional study aimed to examine online communication of Italian health institutions.
110	Pomares TD, Buttenheim AM, Amin AB, Joyce CM, Porter RM, Bednarczyk RA, et al. Association of cognitive biases with human papillomavirus vaccine hesitancy: a cross-sectional study. Hum Vaccin Immunother 2020;16:1018-23	Cross-sectional study aimed to understand the factors that contribute to hesitancy.
111	Powell L, Nour R, Zidoun Y, Kaladhara S, Al Suwaidi H, Zary N. A web-based public health intervention for addressing vaccine misinformation: Protocol for analyzing learner engagement and impacts on the hesitancy to vaccinate. JMIR Res Protoc 2022;11:e38034	Intervention protocol.
112	Reno JE, O’Leary ST, Pyrzanowski J, Lockhart S, Thomas J, Dempsey AF. Evaluation of the implementation of a multicomponent intervention to improve health care provider communication about human Papillomavirus vaccination. Acad Pediatr 2018;18:882-8	Article that develops interventions aimed at healthcare staff to improve communication with hesitant patients.
113	Reuben R, Aitken D, Freedman JL, Einstein G. Mistrust of the medical profession and higher disgust sensitivity predict parental vaccine hesitancy. PLoS One 2020;15:e0237755	Cross-sectional study aimed to understand the factors that contribute to hesitancy.
114	Riddell RP, O’Neill MC, Campbell L, Taddio A, Greenberg S, Garfield H. Featured article: The ABCDs of pain management: A double-blind randomized controlled trial examining the impact of a brief educational video on infants’ and toddlers’ pain scores and parent soothing behavior. J Pediatr Psychol 2018;43:224-33	Analysis article of effective education methods. The objective of this article is how to manage pain among babies and children.
115	Rosen AD, Senturia A, Howerton I, Kantrim EU, Evans V, Malluche T, et al. A COVID-19 vaccination program to promote uptake and equity for people experiencing homelessness in Los Angeles County. Am J Public Health 2023;113:170-4	Analysis article of effective education methods.
116	Ryan GW, Goulding M, Borg A, Minkah P, Beeler A, Rosal MC, et al. Development and beta-testing of the CONFIDENCE intervention to increase pediatric COVID-19 vaccination. J Pediatr Health Care 2023;37:244-52	COVID-19 vaccine intervention.
117	Rzymski P, Borkowski L, Drąg M, Flisiak R, Jemielity J, Krajewski J, et al. The strategies to support the COVID-19 vaccination with evidence-based communication and tackling misinformation. Vaccines (Basel) 2021;9:109	Analysis article of effective education methods.
118	del Prado Sánchez-Molero Martín M, González LS-B, Suárez CL, Blanco CR. Conocimientos, actitudes y acciones de las enfermeras de Atención Primaria ante la vacuna del virus del papiloma humano. Metas de enfermería 2019;22:20-7.	Cross-sectional study aimed to understand the nurses' knowledge about vaccination.
119	Sarah A, Praveen Raj JD, Kompithra RZ, Mathew LG, Angelin S, John HB. Stories to take the edge off pain during immunization for preschoolers: A randomized controlled trial. Am J Occup Ther 2023;77	Analysis article of effective education methods. The objective of this article is how to manage pain among babies and children.

Number	Reference	Reason of exclusion
120	Schnaith AM, Evans EM, Vogt C, Tinsay AM, Schmidt TE, Tessier KM, et al. An innovative medical school curriculum to address human papillomavirus vaccine hesitancy. Vaccine 2018;36:3830-5	Article that develops interventions aimed at healthcare staff to improve communication with hesitant patients.
121	Schulte K, Schierke H, Tamayo M, Hager L, Engehausen R, Raspe M, et al. Strategies for improving influenza vaccination rates in patients with chronic renal disease. Dtsch Arztebl Int 2019;116:413-9	Analysis article to reduce hesitancy that does not use educational methods
122	Schwarzinger M, Watson V, Arwidson P, Alla F, Luchini S. COVID-19 vaccine hesitancy in a representative working-age population in France: a survey experiment based on vaccine characteristics. Lancet Public Health 2021;6:e210-21	COVID-19 vaccine intervention.
123	Sears G, Tutt M, Sabo S, Lee N, Teufel-Shone N, Baca A, et al. Building trust and awareness to increase AZ native nation participation in COVID-19 vaccines. Int J Environ Res Public Health 2022;20:31	COVID-19 vaccine intervention.
124	Shah, Glenn BA, Chang LC, Chung PJ, Valderrama R, Uyeda K, et al. Reducing Missed Opportunities for Human Papillomavirus Vaccination in School-Based Health Centers: Impact of an Intervention. Academic pediatrics 2020;20	Analysis article to reduce hesitancy that does not use educational methods
125	Sondagar C, Xu R, MacDonald NE, Dubé E. Vaccine acceptance: How to build and maintain trust in immunization. Can Commun Dis Rep 2020;46:155-9	Systematic review.
126	Sudharsanan N, Favaretti C, Hachaturyan V, Bärnighausen T, Vandormael A. Effects of side-effect risk framing strategies on COVID-19 vaccine intentions: a randomized controlled trial. Elife 2022;11	Analysis article to reduce hesitancy that does not use educational methods
127	Sun Y, Li B, Li N, Li B, Chen P, Hao F, et al. Acceptance of COVID-19 vaccine among high-risk occupations in a port city of China and multifaceted strategies for increasing vaccination coverage: A cross-sectional study. Risk Manag Healthc Policy 2022;15:643-55	COVID-19 vaccine intervention.
128	Szilagyi PG, Humiston SG, Stephens-Shields AJ, Localio R, Breck A, Kelly MK, et al. Effect of training pediatric clinicians in human Papillomavirus communication strategies on human Papillomavirus vaccination rates: A cluster randomized clinical trial: A cluster randomized clinical trial. JAMA Pediatr 2021;175:901-10	Article that develops interventions aimed at healthcare staff to improve communication with hesitant patients.
129	Taddio A, Coldham J, Logeman C, McMurtry CM, Little C, Samborn T, et al. Feasibility of implementation of CARDTM for school-based immunizations in Calgary, Alberta: a cluster trial. BMC Public Health 2021;21	Analysis article of effective education methods. The objective of this article is how to manage pain among children.
130	Takagi MA, Hess S, Smith Z, Gawronski K, Kumar A, Horsley J, et al. The impact of educational interventions on COVID-19 and vaccination attitudes among patients in Michigan: A prospective study. Front Public Health 2023;11:1144659	COVID-19 vaccine intervention.
131	Thorpe A, Fagerlin A, Drews FA, Butler J, Stevens V, Riddoch MS, et al. Communications to promote interest and confidence in COVID-19 vaccines. Am J Health Promot 2022;36:976-86	COVID-19 vaccine intervention.
132	Ugarte DA, Lin J, Qian T, Young SD. An online community peer support intervention to promote COVID-19 vaccine information among essential workers: a randomized trial. Ann Med 2022;54:3079-84	Article that develops interventions aimed at healthcare staff to improve communication with hesitant patients.
133	Vanderpool RC, Gaysynsky A, Chou W-YS, Tonorezos ES. Using behavioral science to address COVID-19 vaccine hesitancy among cancer survivors: Communication strategies and research opportunities. J Behav Med 2023;46:366-76	Analysis article of effective education methods.
134	Varman M, Sharlin C, Fernandez C, Vasudevan J, Wichman C. Human Papilloma Virus vaccination among adolescents in a community clinic before and after intervention. J Community Health 2018;43:455-8	Article that develops interventions aimed at healthcare staff to improve communication with hesitant patients.
135	Venegas-Murillo AL, Bazargan M, Grace S, Cobb S, Vargas R, Givens S, et al. Mitigating COVID-19 risk and vaccine hesitancy among underserved African American and Latinx individuals with mental illness through mental health therapist-facilitated discussions. J Racial Ethn Health Disparities 2023;10:1358-70	COVID-19 vaccine intervention.
136	Villarreal-Garza C, Vaca-Cartagena BF, Becerril-Gaitan A, Castilleja-Leal F. Letter to Editor, Re: The first report on Covid-19 vaccine refusal by cancer patients in Italy: Early data from a single-institute survey: Educational Webinar about COVID-19 Vaccines in Oncological Patients: A Promising Strategy to Tackle COVID-19 Vaccine Hesitancy. Eur J Cancer 2021;158:189-90	Opinion-based article.
137	Vyas D, Galal SM, Rogan EL, Boyce EG. Training students to address vaccine hesitancy and/or refusal. Am J Pharm Educ 2018;82:6338	Article that develops interventions aimed at healthcare staff to improve communication with hesitant patients.
138	Wawrzuta D, Jaworski M, Gotlib J, Panczyk M. Characteristics of antivaccine messages on social media: Systematic review. J Med Internet Res 2021;23:e24564	Systematic review.
139	Werk LN, Diaz MC, Cadilla A, Franciosi JP, Hossain MJ. Promoting adherence to influenza vaccination recommendations in pediatric practice. J Prim Care Community Health 2019;10:2150132719853061	Article that develops interventions aimed at healthcare staff to improve communication with hesitant patients.

Number	Reference	Reason of exclusion
140	While A. Evidence-based strategies to promote vaccine acceptance. Br J Community Nurs 2021;26:338-43	Analysis article of effective education methods.
141	Wilhelm J, Calvo X, Escobar C, Moreno G, Véliz L, Villena R, et al. Statement from the Immunization Committee of the Chilean Infectious Diseases Society in reference to vaccine refusal and mandatory policy on vaccination. Rev Chilena Infectol 2017;34:583-6	Opinion-based article.
142	Willis DE, Selig JP, Andersen JA, Hall S, Hallgren E, Williams M, et al. Hesitant but vaccinated: assessing COVID-19 vaccine hesitancy among the recently vaccinated. J Behav Med 2023;46:15-24	Cross-sectional study aimed to understand the factors that contribute to hesitancy.
143	Witus LS, Larson E. A randomized controlled trial of a video intervention shows evidence of increasing COVID-19 vaccination intention. PLoS One 2022;17:e0267580	COVID-19 vaccine intervention.
144	Wong CH, Zhong CC, Chung VC, Nilsen P, Wong EL, Yeoh E-K. Barriers and facilitators to receiving the COVID-19 vaccination and development of theoretically-informed implementation strategies for the public: Qualitative study in Hong Kong. Vaccines (Basel) 2022;10:764	Analysis article of effective education methods.
145	Wong LP, Wong PF, AbuBakar S. Vaccine hesitancy and the resurgence of vaccine preventable diseases: the way forward for Malaysia, a Southeast Asian country. Hum Vaccin Immunother 2020;16:1511-20	Cross-sectional study aimed to understand the factors that contribute to hesitancy.
146	Wong WH-S, So H-K, Rosa Duque JS, Tso WW-Y, Chong PC-Y, Kwan MY-W, et al. Impact of a focus education in Zoom on COVID-19 vaccine hesitancy in Hong Kong parents of the preschoolers. Hum Vaccin Immunother 2022;18:2081460	COVID-19 vaccine intervention.
147	Wood S, Pate MA, Schulman K. Novel strategies to support global promotion of COVID-19 vaccination. BMJ Glob Health 2021;6:e006066	Opinion-based article.
148	Young LE, Sidnam-Mauch E, Twyman M, Wang L, Xu JJ, Sargent M, et al. Disrupting the COVID-19 misinfodemic with network interventions: Network solutions for network problems. Am J Public Health 2021;111:514-9	Analysis article of effective education methods.
149	Yousaf M, Hassan Raza S, Mahmood N, Core R, Zaman U, Malik A. Immunity debt or vaccination crisis? A multi-method evidence on vaccine acceptance and media framing for emerging COVID-19 variants. Vaccine 2022;40:1855-63	Analysis article of effective education methods.
150	Zidoun Y, Kaladhara S, Powell L, Nour R, Al Suwaidi H, Zary N. Contextual conversational agent to address vaccine hesitancy: Protocol for a design-based research study. JMIR Res Protoc 2022;11:e38043	Intervention protocol.
151	Zolotarova T, Dussault C, Park H, Varsaneux O, Basta NE, Watson L, et al. Education increases COVID-19 vaccine uptake among people in Canadian federal prisons in a prospective randomized controlled trial: The EDUCATE study. Vaccine 2023;41:1419-25	COVID-19 vaccine intervention.

Table S5. Summary of the characteristics of included studies

Author	Study Type	Sample size Population Country	Vaccine	Intervention	Evaluation	Conclusions
Gagneur, A et al. 2018 [38]	Quasi-experimental design.	2377 mothers from the *Centre hospitalier universtaire of Sherbrooke*, Quebec. Canada.	Diphtheria, tetanus, poliomyelitis, whooping cough *Haemophilus influenzae* type b (Hib), and pneumococcus	**Tailored message**<br>Educational intervention program based on the motivational interviewing (MI) that addressed several pieces of information such as the 6 vaccine-preventable diseases (VPD) at 2, 4, and 6 months of life, the effectiveness of vaccines, the importance of the routine immunization schedule, and the fears and side effects associated with vaccination.<br>The intervention was adapted to each participating family according to the current intention of the parent to vaccinate his/her newborn at 2 months of age.	Comparison between the vaccine coverage (VC) of infants at 3, 5, and 7 months of age in the intervention group and the control group. Also, the feasibility rate of the intervention was analysed (i.e., the proportion of mothers who received the intervention/mothers who accepted to receive the intervention).	The feasibility rate of the educational session was 93% (1243/1329 mothers).<br>The MI session significantly increased the VC of infants by 3.2, 4.9, and 7.3%, and 2.7, 3.8, and 5.7% at 3, 5, and 7 months of age. There was an increase of 15% in vaccine intention of mothers who received the intervention, and a significant increase in VC in infants.<br>Infants of mothers who received the MI session had an 8% higher chance of having a complete vaccination status at the age of 7 months than did children whose mothers did not receive the intervention.
Costantino, C et al. 2019 [24]	Quasi-experimental design	125 HCWs from the Palermo University Hospital. Italy.	Influenza	**Community education**<br>One-hour educational course<br>was administered to the intervention group. The course was focus on the following topics:<br>a) guidelines and recommendations of the Sicilian Health Authorities about influenza vaccination of HCWs; b) description of the vaccine and of vaccination (schedule, site of vaccination, timetable, possible side effects, etc); c) types of influenza vaccines available during the 2016/2017 season.<br>Control group did not undertake any intervention.	Comparison between the influenza vaccination rate in the intervention group (educational group) and the control group in the current campaign (2016/17) and in previous years' campaigns.<br>Data on baseline attitudes and practices were collected from all HCWs enrolled (including intervention and control groups) throughout questionnaire before the educational intervention.	Overall, 43 HCWs out of 125 (34.4%)<br>of the two groups were vaccinated against influenza during 2016/2017 season. 42% of HCWs who decided to attend the educational intervention got vaccinated, while vaccination prevalence in the control group was 31%.<br>The vaccination coverage increase observed during the last two seasons corresponded to +24% in the intervention group and to +18% in the control group.

Author	Study Type	Sample size Population Country	Vaccine	Intervention	Evaluation	Conclusions
Freed-man, T et al. 2019 [25]	Quasi-ex-perimental design	323 grade 7 students from ten schools ser-viced by On-tario. Canada.	HPV<br>Hepatitis B<br>Quadrivalent conjugated meningococ-cal	**Community education**<br>**Media**<br>**New technologies**<br>Based on the CARD™ system (C - Comfort, A - Ask, R - Relax, D - Dis-tract).<br>Experimental schools:<br>1. A multimedia educational lesson consist-ing of information about a) the specific dis-eases vaccines protect against, b) how vac-cines work, possible side effects and vac-cination procedures; and c) instructions for coping with pain, fear and fainting during vaccination.<br>2. A booklet that summarised CARD™ and students wrote down their preferences.<br>3. A booklet for parents with information on diseases, vaccines, and the CARD™ sys-tem.<br>Control schools: No CARD™ system.<br>1. In-class educational lesson with infor-mation as the intervention school without CARD™ booklet and a short question and answer period.<br>2. Same information as the intervention school package except that it excluded the CARD™ brochure for parents.<br>Complementary interventions to reduce fear and anxiety were also provided in organisa-tion and preparation of vaccination clinics and during vaccination process following the recommendations acquired by the CARD system.	Pre- and post-intervention question-naires:<br>• Baseline questionnaires: Knowledge test (about the effec-tiveness of various strategies to reduce pain, fear, and fainting); questions regarding their level of fear of vaccination needles, and willingness to get vaccinations.<br>• Post-presentation questionnaires: Knowledge and attitudes ques-tionnaires that students com-pleted before the intervention. An additional survey about students' opinions of the education session, (questions about level of under-standing, amount of information, usefulness of information, and student level of preparedness for vaccination).<br><br>Focus groups interviews two weeks af-ter the intervention in which students, parents and school staff share their ex-perience of school vaccinations.	The CARD™ system has had a positive im-pact on students' knowledge, attitudes, use of coping strategies and some symptoms of vaccination. Students reported that they un-derstood the information and that it pre-pared them for vaccination: Knowledge scores were higher post education (P<0.001). Student level of fear was lower (P=0.03). There was an increase in willing-ness to be vaccinated (P=0.001).<br>These facts are confirmed by the interviews conducted in the focus groups: Experi-mental school students reported increased vaccine-related knowledge after the pre-vaccination education session. Students felt that CARD™ helped prepare them for vaccinations.

Author	Study Type	Sample size Population Country	Vaccine	Intervention	Evaluation	Conclusions
Kim, M et al. 2019 [52]	Randomized controlled trial	104 university Korean female students who resided in the North region. USA.	HPV	**New technologies** The intervention group received a storytelling video that engages two narrators in an interactive conversation and narrates HPV-related vaccination stories, allowing for expanded commentary with scientific and evidence-based information on HPV, the HPV vaccine and cervical cancer by a Korean American doctor. Comparison group received written HPV vaccine information from the American Cancer Society and the CDC, which takes about 10–15 min to read completely	Acceptability of the intervention was assessed for both groups with five questions related to satisfaction and approval of the educational programme: (Q1) Satisfaction with the educational programme (Q2) Increase in their knowledge about HPV, HPV vaccine and cervical cancer (Q3) Change in their perceptions about the vaccine (Q4) Change in their feelings about getting the vaccine (Q5) Decision to get the HPV vaccine. Response options used a five-point Likert scale.	Video intervention resulted in significantly greater satisfaction with the intervention and more positive feelings about getting the HPV vaccine when compared with the text-based comparison group ($P < 0.05$). Compared with the comparison group, participants in the storytelling intervention group had significantly greater satisfaction with the intervention (Q1) and the intervention had a significantly greater influence on their feelings about getting the HPV vaccine (Q4) ($p < 0.05$).
Blanchard, J-L et al. 2020 [26]	Quasi-experimental design. Pre-post study.	598 high school students between 13 and 18 years from the North Bay Ontario. Canada.	Vaccine-preventable diseases (VPDs) recommended from Canadian Pediatric Society.	**Community education** Educational intervention which focused on three concepts: Describing the risks that vaccine-preventable diseases can carry (showing the diseases with videos and interviews with people who had contracted them, providing general information about the diseases); the benefits of vaccination (examples of herd immunity and interactive presentations and scenes around vaccine reluctance) and the challenges of communicating information about vaccines to adolescents (most common reasons for refusing immunisation, and a discussion on how to counteract vaccine hesitancy).	An anonymized questionnaire was administered to assess students' knowledge, attitudes and beliefs about immunizations and VPDs on the same day before and after delivering the education session.	Students felt they were more informed about immunizations and the diseases they prevent with a median agreement increase from 6 (neutral) to 8 (agree) ($P < 0.001$). The intervention improved the students' perception that immunizations are important to their health (median increase in agreement from 6 (neutral) to 8 (agree), ($p < 0.001$)). The intervention also appears to have influenced the students' motivation to encourage their friends and family to be immunized (median increase in agreement from 5 (neutral) to 7 (agree), ($p < 0.001$)).

Author	Study Type	Sample size Population Country	Vaccine	Intervention	Evaluation	Conclusions
Glanz, J. M et al. 2020 [39]	Randomized controlled trial	824 pregnant women and new parents of Denver and Boulder. USA	General vaccinations in childhood	**Tailored message**<br>**New technologies**<br>Participants were randomly assigned to 1 of 3 study arms:<br>Web-based tailored messaging "Vaccines and Your Baby" intervention (VAYB):<br>The website included surveys, one-on-one interviews, usability testing and messages that conveying the information were tailored to each participants' intention to vaccinate, personal attitudes about vaccination and vaccination values.<br>Untailored version of the intervention (UT): This web had same design and information as the VAYB Web site, but it was not personalized to the participants' survey responses, and the content did not change across the time points.<br>Usual care (UC): Scheduled 20-minute well-child visits at 2, 4, 6, and 12 months of age, with an option for a 9-month visit.<br>The intervention was administered at 4 time points across the follow-up: the last trimester of pregnancy or child age <2 months, at child aged 4 to 6 months, at child aged 10 to 12 months, and at child aged 13 to 15 months.	The primary outcome was immunization status assessed over the first 200 days of age to cover a majority of the routinely administered infant vaccines and to minimize loss to follow-up.	Provision of a Web-based tailored messaging intervention to expectant and new parents did not have a positive effect on the timely uptake of infant immunizations.<br>The proportions of infants who were up to date at age 200 days were 91.4%, 92.9%, and 92.3% for the VAYB, UT, and UC arms, respectively.<br>Infants in the VAYB were not more likely to be up to date than infants in the UC arm (OR = 0.89; 95% CI, 0.45–1.76), nor were they more likely to be up to date than infants in the UT arm (OR = 0.82; 95% CI, 0.42–1.63). Similarly, the odds of being up to date did not differ between the UT and UC arms (OR = 1.08; 95% CI, 0.54-2.18).
Icardi, G et al. 2020 [27]	Literature review and pre-post study	1.160 pre-adolescents and 161 parents of pre-adolescents. Italy.	HPV	**Community education**<br>**Media**<br>Educational session in the school setting for pre-adolescents conducted by the research group and a written text for the pre-adolescents' parents	Comparison between questionnaire before and after educational session about participant's general characteristics, knowledge about HPV infection or HPV vaccination and the willingness of the participant to get vaccinated.	The first group obtained an average score of 8.59 ± 1.70 (CI 95% 8.49 - 8.69) and 9.10 ± 1.43 (CI 95% 9.01 – 9.19) at the pre- and post-educational intervention, showing a statistically significant increase (p<0.0001). The median pre-intervention score estimated in parents was 10 (25-75p = 8-10) and remained high after providing written information (median, 25-75p = 10, 7.8-10).

Author	Study Type	Sample size Population Country	Vaccine	Intervention	Evaluation	Conclusions
Johri, M et al. 2020 [28]	Randomized controlled trial	391 caregivers of children aged 0 to 12 months from rural villages in a district of Uttar Pradesh. India.	General vaccination in childhood	**Community education**<br>**New technologies**<br>Development of a social and behaviour change communication (SBCC) that were delivered through 2 channels (1) mobile health (mHealth): entertaining educational audio capsules (edutainment) by push messages (automated dial outs) and voice immunization reminders via mobile phone and (2) face-to-face: community mobilization activities, consisting of 1 large introductory meeting to introduce the project to communities and 3 small meetings offered to each participant covering specific themes.<br>The control group received usual services.	– Face-to-face surveys: Interviewer-administered household surveys were conducted. Surveys were administered before random group assignment and approximately 5 months later following interventions at end line.<br>– Project administrative records: Standardized forms to assess delivery of study procedures and interventions were maintained by field staff.<br>Interactive voice response (IVR) system: The IVR system automatically recorded all calls to the platform.	Immunization knowledge was low at baseline in both study groups. For 3 of the 4 indicators studied, knowledge improved in the intervention group at end line. Differences-in-differences estimates of impact suggest that observed improvements were owing to the study interventions.<br>For 8 of 9 intermediate outcomes, the regression results showed significantly higher basic health knowledge among the intervention group at end line.
Nowak, G. J et al. 2020 [53]	Randomized controlled trial	171 influenza vaccine avoidant 18-49-year-old adults in Georgia. USA.	Influenza	**New technologies**<br>In the control arm, a Vaccine Information Statement (VIS) was developed to provide neutral and balanced benefit-risk information in a non-persuasive format.<br>Three intervention arms were developed and included VIS and one of the following interventions:<br>• VR: Participants wore a head-mounted display unit and experienced a five-minute VR story that included use of interactive video game controllers as part of the immersive mediated environment.<br>• Video: Participants viewed the same content as the VR condition through a desktop monitor<br>E-pamphlet: Participants used a computer tablet to view the same information conveyed with text and static images from the video.	Participants completed a pre- and post-intervention survey.<br>The pre-test survey included general questions about whether they had ever had the disease, whether a doctor advised them to get vaccinated, and whether they should get vaccinated now.<br>The post-test survey contained (1) measures of three potential mediating variables - presence, concern about spreading flu to others, and empathy and (2) measures for seven dependent variables – community immunity beliefs, flu susceptibility, self-flu severity, other-flu severity, flu vaccination confidence, confidence that one's flu vaccination protects others, and flu vaccination intention.	VR technology and the ways it can impact information processing have the potential to increase targeted audience understanding of key vaccination concepts.<br>The findings here suggest that vaccination education, particularly when targeting vaccination-avoidant adults, may also yield greater impact when information is conveyed through more immersive media platforms that enable users to experience key concepts through multiple layers of sensory cues.<br>VR's ability to positively influence key psychological variables, such as the perception of presence or indirectly, the concern for others' well-being driving the positive effects.

Author	Study Type	Sample size Population Country	Vaccine	Intervention	Evaluation	Conclusions
Davies, C et al. 2021 [29]	Randomised controlled trial	6967 students from 40 schools. Australia.	HPV	**Community education**<br>**Media**<br>**New technologies**<br>Intervention schools delivered a multicomponent education and logistical intervention comprising 1) an adolescent intervention: education taught through the school in an interactive lesson (18 min animated film), a take-home magazine designed by and for adolescents, online components that can be accessed outside the school in a website and app for mobile devices, and distraction/relaxation methods to be used prior to and during vaccination; 2) an HPV vaccine parent/adolescent decision support tool: ; and 3) logistical strategies: methods for increasing consent form return such as direct mail-out of forms to parents, reminders, and non-material incentives.<br>Control schools conducted the vaccination program as per their usual practice.	The questionnaire HAVIQ was developed and validated to collect data in 4 subscales: (1) knowledge about HPV and the HPV vaccine (6 items), (2) adolescent involvement in decision-making (8 items), (3) fear and anxiety associated with the vaccine (6 items), and (4) self-efficacy in receiving the vaccine (5 items).<br>Intervention impact was evaluated using a pretest and post-test of mean difference in scores for each relevant subscale on the measure, between intervention and control groups.	Students in intervention schools had, on average, 65% correct responses to the HAVIQ knowledge questions, which were administered pre-HPV dose 1 of vaccine. This compared to 33% of responses correct by students in control schools; this was highly statistically significant.<br>Intervention improved adolescent psychosocial outcomes, although the overall differences were small:<br>The mean score before dose 1 for decision-making on the HAVIQ subscale 2 was 3.50 (0.42) of 5 points in the intervention group and 3.40 (0.40) in the control group, a small but statistically significant difference. There was a small reduction in vaccination-related anxiety and a small increase in vaccination-related self-efficacy in the intervention group compared with the control group.
Di Mauro, A et al. 2021 [54]	Quasi-experimental design. Pre-post study	170 preterm infants. Parents of preterm infants. Italy.	Hexavalent, Meningococcus C and B, Pneumococcus, Rotavirus and Tetravalent.	**New technologies**<br>On NICU Facebook page, the research team posted short and easy-to-read contents, with information about risks and benefits of vaccines, vaccine-preventable diseases and the recommended immunization schedule. Parents were allowed to post comments and questions and to get answers from the team.	Comparison of immunization rates of the 2016-2017 intervention cohort with a historical cohort of preterm infants (2013-2014) not exposed to web-based interventions and the regional paediatric population cohort (2016-2017).	Vaccination rates of preterm infants were not significantly different from those of regional general paediatric population ($p > 0{,}05$).<br>DTaP-IPV-HBV-Hib 2 and 3 doses, MMR and Varicella shows higher values in vaccine coverage in 2016–17 preterm cohort than historical preterm cohort ($p < 0.05$).
Lecce, M et al. 2021 [55]	Quasi-experimental design. Pre-post study	Healthcare workers of a hospital in Milan. Italy.	Influenza	**New technologies**<br>The promoting and educational campaign was conducted on the intranet platform of the hospital, gaming strategy in which the hospital is divided by department and the department with the highest vaccination rate wins and the delivery of flu vaccines through both an ad hoc ambulatory and several on site vaccination teams.	Comparison between the seasonal influenza vaccination rate and the rate in previous years in which the first 2 strategies mentioned above were not implemented.	In the 2020-2021 influenza vaccination campaign for HCWs, 2103 healthcare workers were vaccinated, reaching a comprehensive vaccination coverage of 43.1%. This compared to 1153 vaccinated HCWs (VCR 21.5%, +82,4%) in the 2019-2020 campaign and 759 vaccinated HCWs (VCR 14.5%, +165,2%) in the 2018-2019 campaign.

Author	Study Type	Sample size Population Country	Vaccine	Intervention	Evaluation	Conclusions
Surya-devara, M et al. 2021 [46]	Quasi-experimental design. Pre-post study	31.408 adolescents from different counties of the New York State. USA.	HPV	**Media**<br>Distribution of cancer prevention booklets, that they were developed to combine HPV vaccine information with other routinely provided cancer prevention guidance, to de-emphasize the association of HPV vaccine and sex, while re-framing the message of HPV vaccine and cancer prevention, with a goal of increasing adolescent HPV vaccine uptake.	Comparison between practice-specific HPV vaccination rates before the education session and 12 months later.<br>In addition, comparison these numbers with the county-wide HPV vaccination rates for the same start and end dates.	Aggregate practice data show that 1 year after program start, HPV vaccine series initiation rates in both the 11–12-year (33% versus 40%) and 13–18-year (70% versus 73%) cohort and HPV vaccine completion rates in the 13–18-year cohort (56% versus 59%) were significantly higher than baseline.
Yousuf, H et al. 2021 [56]	Randomized controlled trial	980 elderly citizens in Netherlands.	General vaccination	**New technologies**<br>The outcomes of the diagnostic survey were used to design videos aiming at the gaps in understanding and misconceptions surrounding vaccinations.<br>In both videos three TV celebrity scientists were displayed, explaining the different aspects of vaccination, including social norm, information on vaccinations.<br>The control video (Video 1) contained only information on vaccination and social norms (Non-debunking video): (1) groups at higher risk, (2) the effect of healthy lifestyle behaviour, (3) the safety, effectiveness, working mechanisms and development of vaccines, (4) the safety and contents of the influenza vaccine, and that (5) GP practices have taken effective precautions against COVID-19 to ensure safety of individuals receiving an influenza vaccine<br>Video 2 contained all the contents of Video 1, but on top also had several debunking fragments on vaccination misconceptions: (1) the safety, efficacy and development of vaccines in general, (2) the falsely proclaimed link between autism and vaccines, (3) the safety and contents of the influenza vaccine.	Pre- and post-intervention questionnaire covering the following topics: (A) governmental trust on influenza vaccination (7 questions, adapted from WHO SAGE), (B) vaccine hesitancy (10 questions, adapted from a governmental trust survey during the H1N1 pandemic), and (C) myths and knowledge about influenza and COVID-19 (6 questions).	The debunking strategies were effective in increasing knowledge and awareness surrounding vaccinations, combatting vaccine misinformation, and enhanced the trust in governmental institutions.<br>The debunking strategy, on top of social norm and vaccine information, helped reject misconception that vaccination caused autism, weakened the immune system, and that the influenza vaccination adversely affected the efficacy of COVID-19 vaccination.<br>Intriguingly, exposure to the debunking video also improved the knowledge pertaining to the effect of influenza vaccination in preventing cardiovascular events more.

Author	Study Type	Sample size Population Country	Vaccine	Intervention	Evaluation	Conclusions
Bethke, N et al. 2022 [30]	Randomized controlled trial	863 grades 9 to 11 students from four schools of Berlin. Germany.	MMR and Tdap-IPV	**Community education** **New technologies** Two planned intervention conditions were tested, ie, the Educational Class Condition (ECC) and Low-Intensity Information Condition (LIIC). <u>ECC</u>: 90-minute procedure that included an educational unit (45 min) consisted of a presentation about the immune system and infectious diseases, vaccination processes, herd immunity, and the risks and benefits specific to measles vaccination. A group discussion as well as media elements such as newspaper articles and videos were also included. The educational unit was based on three theories of health behaviour change: SCT, PMT, and the HBM. <u>LIIC</u>: 45-minute unit providing information about the Prevention Bus and the vaccines offered, as well as information about the procedures.	• Post-intervention vaccination rate in the Prevention Bus.<br>• Vaccination-related knowledge scale indicated the level of immunization knowledge.<br>• Further feedback was recorded with semi structured interviews in the educational unit.<br>• The vaccination-related perceived self-efficacy scale reflected students' beliefs about their own competency to understand, appraise, and apply vaccination-related health information.<br>The scales were administered either before and after the educational interventions or only after them.	The vaccination-related knowledge, the percentage of correct responses before the educational unit ranged from 22% (CI 16%/28%) to 70% (CI 65%/76%). After the educational unit, these rates increased to 32% (CI 23%/40%) to 86% (CI 81%/92%). Looking at the post-intervention results, a significant difference (B=-0.64, CI: -0.94/-0.33, p<0.001), indicating a higher knowledge sum score after the educational unit (2.98, CI: 2.78/3.17) than after the basic information (2.34, CI: 2.15/2.54). The results showed that the school-based on-site vaccination approach is a feasible way to improve vaccination rates. Students in all participating schools showed a good vaccination uptake rate after the pilot intervention and provided good feedback regarding the educational components of the intervention.
Boccalini, S et al. 2022 [31]	Quasi-experimental design. Pre-post study.	449 students from degrees in Medicine and Surgery, in Pharmacy, and in the postgraduate school of Hygiene and Preventive Medicine of the University of Florence. Italy.	Vaccine-preventable diseases and specifically COVID-19	**Community education** An Elective Teaching Activity (ETA) on vaccines and vaccinations was organized through online platform Webex and Moodle. The course (16 h) included lessons on the different types of vaccines, preclinical and clinical trials, regulatory process, process of vaccine production, supply, and storage, dispensation of vaccines, Health Technology Assessment (HTA) of new vaccines or vaccination strategies, national immunization plan, immunization coverage, impact of current immunization strategies, pharmacovigilance on vaccines and, lastly, fake news and scientific evidence on vaccines. A relevant focus was dedicated to development and authorization of COVID-19 vaccines, their characteristics and safety profile.	Pre- and post-intervention questionnaire comparison. Questionnaires contained 30 multi-choice questions on the main topics of the teaching activity.	The results obtained suggest that the ETA was highly effective in increasing the students' knowledge on vaccination: despite good overall scores in the pre-course test, the different groups were able to increase their final score by 27.3% (p < 0.001; average improvement for individual students 26.59%).

Author	Study Type	Sample size Population Country	Vaccine	Intervention	Evaluation	Conclusions
Keselman, A et al. 2022 [47]	Randomized controlled trial	311 students from the Lucerne University of Teacher Education. Switzerland.	HPV	**Media**<br>Adapted brochure about HPV vaccination developed using a US CDC brochure.<br>The standard brochure included the following sections: What is HPV? What is HPV vaccination? How effective are the HPV Vaccines? Are HPV vaccines safe?<br>The extended version of the brochure included an in-depth biological explanation of the nature of viruses and the HPV vaccine, as well as the mechanism by which the HPV vaccine interacts with the immune system.	Participants completed the same questionnaire before and after intervention where they were asked to rate ten statements on a 5-point scale.<br>the statements assessed each of the following variables: Susceptibility to HPV Infection; importance of HPV Vaccination; safety of HPV Vaccination; effectiveness of HPV Vaccination.	Participants in both groups showed a significant positive change in their beliefs about vaccination safety, effectiveness, and importance in preventing cervical cancer. A single read of a standard brochure may positively impact some individuals' beliefs about HPV vaccination.<br>Contrary to our hypothesis, adding biology-enhanced text to the standard brochure did not have an overall positive effect on participants' beliefs about HPV vaccination safety. However, the biological data had a greater impact among the vaccination-neutral population.
Lewin, E. B et al. 2022 [57]	Randomized controlled trial	47 7th grade students enrolled in the Montgomery and Virginia public school systems. USA.	General vaccinations in adolescents (pertussis, meningococcus, influenza, and HPV)	**New technologies**<br>Intervention group: Students in this group completed Vaccine Education Program (VEP), consists of thirteen videos, covering topics about vaccine development, herd immunity, and topic-specific information about vaccines administered to adolescents.<br>Control group: Students saw National Geographic video called “Chemistry of Cells” which contained detailed information about the biology, structure, and life cycle of cells with no reference to vaccines or the immune system.	Students completed a pre- and post-intervention questionnaire containing:<br>• 10 knowledge questions with multiple-choice response options reflected core concepts about vaccines and immunity contained in the VEP vaccine videos.<br>• 6 beliefs and attitude questions about natural immunity, the protective value of vaccines and safety concerns.<br>5 satisfaction questions asked participants in the intervention group.	The program was successfully delivered, and results showed favourable shifts in vaccine-related beliefs and high satisfaction with program content.<br>Both groups had higher post-test knowledge scores compared to pre-test values. The vaccine group had greater improvement than the comparison group; however, there were no statistically significant differences (p=0.77).<br>There was more than a two-fold shift toward positive vaccine beliefs and attitudes in the vaccine compared to the comparison group (27.5% vs. 12.1%, p<0.01) and a greater proportion toward more negative vaccine-related beliefs and attitudes in the control group than in the vaccine group, but the difference was not statistically significant (9.8% vs. 5.9%, p=0.34).

Author	Study Type	Sample size Population Country	Vaccine	Intervention	Evaluation	Conclusions
Muñoz - Miralles, R et al. 2022 [40]	Randomized controlled trial	524 individuals in high-risk groups for influenza and not intending to be vaccinated against the influenza virus in Catalonia.<br>Spain.	Influenza	**Tailored message**<br>In the intervention group, patients were asked about the reasons to reject the inactivated influenza vaccine (IIV). The intervention consisted of a standardized brief intervention, performed by the healthcare professional, which varied depending on the reason the patient had given for refusing the vaccination.<br>In the control group the IIV advice was the normal and unstructured advice that professionals used to give their patients, that they do every year.	Comparison between the influenza vaccination rate in the intervention group (standardized brief intervention) and the control group (normal advice) at the end of the IIV campaign.	At the end of the IIV campaign, 75 patients (29.9%) in the IG were vaccinated and 40 in the CG (14.6%). The intervention was effective at the global level (odds ratio [OR]: 2.48 [1.61-3.82]; $p<0.001$) and in those aged 60 and over 60 (healthy OR: 2.62 [1.32-5.17], and with risk factors OR: 2.95 [1.49- 5.79]). There were no statistically significant differences in the effectiveness of the intervention in those aged under 60 with risk factors or between individuals with different illnesses.
Shegog, R et al. 2022 [58]	Randomized controlled trial	512 parents of vaccine-eligible youth (10- 17 years old) in Houston.<br>USA.	HPV	**New technologies**<br>The intervention group to use the HPVcancerFree (HPVCF) app in addition to usual care by health professionals. HPVCF comprises four components: 1) 'HPV A-Z' (a compendium of content domains providing facts about HPV and HPV vaccine), 2) 'Bust A Myth' (educational modules including peer and provider testimonials addressing the most salient HPV vaccination barriers); 3) 'Notes 4 Doc' (a medium to facilitate communication with providers on HPV vaccine), and 4) 'Get the Vax' (enabling parents to schedule tailored HPV vaccination appointment reminders).<br>The control group had only usual care.	Baseline (pretest) and 5-month follow-up (post-test) surveys assessed vaccination initiation rates and psychosocial predictors of vaccination:<br>– Vaccine initiation: Proportion of the study participants who reported that their vaccine-eligible child received at least one HPV vaccine (initiated) between the pre-survey and completion of the post-survey.<br>Psychosocial predictors of vaccination behaviour: Intention to vaccinate, knowledge, perceived effectiveness, outcome expectations, perceived susceptibility, barriers, and norms, perceived future regret, and self-efficacy.	Digital behavioural change interventions like the HPVCF influence parent decision-making and initiation of HPV vaccination.<br>Parents in the treatment (HPVCF) condition demonstrated significantly improved knowledge about HPV and the HPV vaccine ($p < .05$) as well as perceived effectiveness of the HPV vaccine ($p < .05$) compared to parents in the comparison condition.<br>HPV vaccination initiation rates were not significantly different between study conditions.<br>Those parents also demonstrated higher initiation of Tdap and MCV, greater knowledge about HPV-related disease, and more positive perceived norms though differences were not statistically significant.

Author	Study Type	Sample size Population Country	Vaccine	Intervention	Evaluation	Conclusions
Taddio, A et al. 2022 [32]	Randomized controlled trial	1919 grade 7 students from ten schools serviced by Ontario. Canada.	HPV Hepatitis B Quadrivalent conjugated meningococcal	**Community education** **Media** **New technologies** Based on the CARD^{TM} system (C - Comfy - Ask, R - Relax, D - Distract). Intervention schools include education of students: In-class lesson about CARD (including 2 videos and handout) with distribution of vaccination consent forms and consent for use of topical anesthetic and student selection of coping interventions; education of staff providing vaccination services; clinic planning with principal; reminders; clinic set-up and processes and interactions during vaccination. Control schools followed usual practices, which included confirmation of vaccination clinic appointment times and delivery of vaccinations on clinic day.	Comparison of vaccination rates between control and intervention schools. During each vaccination interaction, nurses completed a checklist that documented procedural data, coping interventions used and faints and other post-vaccination stress-related responses. Immediately after vaccination, students independently self-rated fear, pain, and dizziness during vaccination (0–10) and perceptions about whether they should get vaccinations (5-point Likert scale), and for CARD schools, how much CARD helped and willingness to use CARD in the future. Staff knowledge and attitudes were collected from surveys, checklists, and a facilitated focus group discussion held after clinics	CARD reduced student stress-related responses and was acceptable and feasible in this real-world setting. Fewer students in CARD schools experienced fear compared to controls: OR 0.65 (95% CI 0.47–0.90). Similarly, pain was lower in CARD schools compared to control schools: OR 0.62 (95% CI 0.50–0.77). There were 0 (0%) vs. 6 (0.8%) episodes of fainting in CARD vs. control schools ($p = 0.02$), with each faint occurring in a different school (i.e., not clustered). Dizziness and post-vaccination reactions did not differ. The overall vaccination rate, although higher in the CARD schools (76.1%) compared to control (72.5%), was not statistically significant; OR 1.13 (95% CI 0.85–1.50).
Zhang, X et al. 2022 [33]	Randomized controlled trial	946 female freshmen from two universities of Sichuan and Shanxi. China.	HPV	**Community education** The intervention group received a 10-min daily web-oriented multicomponent education for one week, whereas the control group received some health tips about COVID-19, which was not relevant to HPV vaccination. The intervention covered the accompanying themes: (1) prevention of vaccination and sexual health; (2) information on HPV infection and its risk factors; (3) presentation of HPV immunization; (4) case study showing the prognosis of Chinese women with advanced cervical cancer; (5) storytelling the experience; (6) information to improve self-efficacy and self-determination; and (7) social strategies for HPV vaccination. Students from control group were provided with educational materials unrelated to HPV prevention during the same period.	Three self-administered electronic questionnaires (before intervention, immediately after the intervention and one month after the intervention) to assess: (1) general information about the study participants; (2) perceptions of sexual health education; (3) knowledge of HPV and HPV vaccine; (4) willingness to receive the HPV vaccine.	After the intervention, awareness of HPV, HPV-related diseases, and HPV vaccines in the intervention arm were significantly higher than control arm ($p < 0.001$). In addition, the knowledge score regarding HPV and HPV vaccines was considerably higher in the intervention group than that in the control groups (5.13 ± 1.23 vs. 3.10 ± 1.99, $p < 0.001$). However, the correct rates of "HPV infection is almost asymptomatic" (21.2%) remained low. After the intervention, willingness to be vaccinated and desire to encourage others to take HPV vaccines increased from 36.3% to 45.6% and 71.2% to 84.4%, respectively, in the intervention group. However, the number of vaccinated students remained low, with only 1.6% and 1.8% in the control and intervention groups, respectively.

Author	Study Type	Sample size Population Country	Vaccine	Intervention	Evaluation	Conclusions
Salmon, D. A et al. 2019 [41]	Quasi-experimental design. Pre-post study	1103 pregnant women from 23 obstetrician-gynecologist offices in Georgia and Colorado. USA	Maternal and infant vaccines.	**Tailored message** **New technologies** MomsTalkShots is an individually tailored educational application (app) that delivers algorithmically tailored videos based on parent needs, vaccine attitudes, beliefs and intentions. The videos address vaccine safety, serious side effects, timing of vaccines, and vaccine ingredients. The intervention is rooted in theoretical behavioural constructs, including risk perception, vaccine efficacy, empathy, and self-efficacy, and is guided by the Elaboration Likelihood Model (ELM) for persuasive communication and behaviour change.	Pre- and post-intervention questionnaire comparison. The pre-post intervention survey included questions related to socio-demographics, intention to receive maternal vaccines (such as Tdap and influenza) and infant vaccines, confidence in vaccination, and self-assessment of knowledge about vaccines before and after watching the intervention videos, to evaluate the impact of the intervention on participants' perceived knowledge. Also asking about information needs and usability of intervention.	Most women reported that MomsTalkShots was helpful (95%), trustworthy (94%), interesting (97%), and clear to understand (99%). Among women who did not have enough vaccine information before watching the intervention videos, the majority reported having enough information after watching them (72%).
Zhang, X et al. 2020 [34]	Quasi-experimental design.	5,024 adolescents aged 13 to 14 years from 14 middle schools. China.	HPV	**Community education** The intervention group received a 45-minute health education session delivered by trained teachers. The session consists in a PowerPoint-oriented lecture on HPV and HPV vaccines. The control classes were not offered the health education lecture.	Pre- and post-intervention questionnaire comparison. The baseline questionnaire consisted of socio-demographic characteristics, knowledge about HPV and HPV-related diseases, and students' willingness to be vaccinated. The post-intervention and one-year follow-up questionnaire consisted of questions related to HPV vaccine knowledge and willingness to be vaccinated.	Health education intervention significantly increased HPV-related knowledge among students in the intervention classes. However, there was a decrease in knowledge levels after one year, although it remained higher in the intervention classes compared to the control classes (p < 0,001). Additionally, students in the intervention classes were more likely to vaccinate themselves compared to the control classes one year after the intervention (74.2% vs. 63.7%, p <0,001).

Author	Study Type	Sample size Population Country	Vaccine	Intervention	Evaluation	Conclusions
Dudley, M et al. 2021 [42]	Randomized controlled trial	277 close contacts of pregnant women in Colorado and Georgia. USA	Influenza Dtap	**Tailored message**<br>**New technologies**<br>This study used the MomsTalkShots educational intervention described in the article by Salmon, D.A. et al (2019).<br>The participants were divided into three groups:<br>**1) Intervention group 1**: received the app that participants used to view educational videos and receive and incentive,<br>**2) Intervention group 2**: received a limited version of the app that allowed participants to receive the incentive but did not provide access to the educational videos.<br>**3) Control group:** was not exposed to the app.	Pre- and post-intervention questionnaire.<br>The baseline survey included a multiple-choice question assessing intention to receive recommended vaccines, whereas the follow-up survey included a multiple-choice question assessing the receipt of these vaccines.<br>In addition, each survey included Likert scale statements assessing confidence in vaccine safety and efficacy, perceived risk of vaccine-preventable diseases (VPDs), descriptive and injunctive social norms, self-efficacy, perception of knowledge, and trust in information sources.	After watching the individually tailored educational videos on the app, there were significant increases in influenza vaccine knowledge ($p = 0.025$), Tdap vaccine knowledge ($p < 0.001$), and intention to receive these vaccines ($p = 0.046$).<br>Of the participants who completed the follow-up survey, 33% reported receiving both influenza and Tdap vaccines, 41% reported receiving only influenza vaccine, 3% reported receiving only Tdap vaccine, and 24% reported receiving neither vaccine.<br>The provision of small pharmacy-based financial incentives combined with individually tailored educational videos led to 6.97 times higher odds of self-reported receipt of influenza vaccine compared to providing small pharmacy-based financial incentives without the videos.
Ibraheem, R et al. 2021 [48]	Randomized controlled trial	560 caregiver or mother whose infants are about to start the National Immunization Program in Ilorin. Nigeria.	BCG, hepatitis B virus, oral polio, pentavalent, pneumococcal conjugate, inactivated polio, measles and yellow fever vaccines.	**Media**<br>The participants were divided into 4 groups:<br>**A) Call reminders.**<br>**B) SMS reminders.**<br>**C) SMS health education**: Messages about the importance of vaccination and information on vaccination schedules and appointments.<br>**D) Routine care** (Control group).	Comparison of the appropriateness of the timing of presentation/ receipt of vaccination dose for the five series of vaccinations scheduled, and the vaccination completion rates between interventions group and control group.	The call reminder group had the highest odds of presenting for immunization, followed by the SMS reminder group and the SMS health education group.<br>The vaccination completion rates were high across all intervention groups, with over 96% of children completing vaccination by the fifth visit.<br>However, the control group had a lower completion rate, with only 90.4% of children completing vaccination by nine months of age.

Author	Study Type	Sample size Population Country	Vaccine	Intervention	Evaluation	Conclusions
Suzuki, Y et al. 2021 [59]	Randomized controlled trial	1660 participants aged 20 years or older in Tokio. Japan.	HPV	**New technologies** The sample was divided between those participants who answered the questionnaire after (intervention group) or prior to (control group) providing behavioural insights material (BI-material) featuring brief scientific information presented in an easy-to-read format. The behavioural insights (BI) material contained information on the prevalence and impact of cervical cancer in Japan, emphasizing the need for vaccination.	Comparison of the questionnaire between the control group and the intervention group. The questionnaire consisted of 10 items, with the first half focusing on HPV awareness as background information and the second half addressing willingness to receive HPV vaccines and undergo screening tests.	Providing brief scientific information could increase the willingness to consider HPV vaccination for daughters and sons. 29.2% of the respondents were aware of the benefits of HPV vaccination. The intervention group showed a higher willingness for immunization of their daughters compared to the control group (aOR 1,32, IC 95% 1,04-1,69). The intervention group also had significantly higher awareness of the benefits and adverse events associated with the HPV vaccine compared to the control group (by 6.5% and 6.6%, respectively).
Dudley, M. Z et al. 2022 [43]	Randomized controlled trial	2087 pregnant women from diverse prenatal care practices in Colorado and Georgia. USA.	Maternal and infant vaccines	**Tailored message** **New technologies** This study used the MomsTalkShots educational intervention described in the article by Salmon, D.A. et al (2019). After the administration of the initial surveys, the study population was divided into the intervention group (received access to the app) and the control group (did not receive anything).	Comparison of the questionnaire between the control group and the intervention group at baseline of study, 1 month and 1 year after their infant's birth. Surveys used multiple choice questions to assess vaccine intentions, and Likert scale statements to assess vaccine knowledge, attitudes, and beliefs.	By 1-month post-birth, the app increased perceived risk of maternal influenza disease, confidence in efficacy, and perceived knowledge. By 1-year post-birth, MomsTalkShots increased perceived vaccine knowledge and trust in vaccine information from obstetricians and paediatricians.
Lubis, T. A et al. 2022 [60]	Quasi-experimental design.	220 parents with children aged 10-18 month in North Jakarta. Indonesia.	General childhood vaccination	**New technologies** The intervention group received the educational videos less than 2 minutes distributed via WhatsApp that included five modules covering topics such as the danger and prevention of vaccine-preventable diseases, rationales for completing immunization, immunization during the COVID-19 pandemic, vaccine misconceptions, and adverse events following immunization and how to treat them. The control group was given exposure to the digital version of the MCH handbook, consisting of animated pictures and simple instructions	Comparison of the pre- and post-intervention vaccine hesitancy survey between the control group and the intervention group. The questionnaire used was Parent Attitudes about Childhood Vaccines (PACV) that quantify vaccine hesitancy, consists of 15 questions, where higher scores indicate higher levels of vaccine hesitancy.	There is a significant difference in post-intervention PACV median score between the intervention and the control group, indicating significantly lower vaccine-hesitant parents in the intervention group after watching videos.

Author	Study Type	Sample size Population Country	Vaccine	Intervention	Evaluation	Conclusions
Suzuki, Y et al. 2022 [61]	Randomized controlled trial	2.175 parents aged with a daughter 11 and 18 years of age. Japan.	HPV	**New technologies**<br>The intervention group received a short film on a cervical cancer survivor to the participants in the intervention group.<br>She talked about her experience—from the beginning of the diagnosis to the sequelae of first-line therapy, while seven subtitle messages on cervical cancer incidence, prevention and screening were inserted throughout his talk.<br>The control group did not receive any intervention.	7-item awareness questionnaire to determine HPV awareness as background information before intervention.<br>After intervention, the questionnaire also assessed attitudes toward HPV vaccination and awareness regarding the prevention of cervical cancer.<br>3 months after the intervention, they asked them if her daughter had received the vaccine or if she was considering vaccination in the future.	The intervention group showed a higher willingness for HPV vaccination compared to the control group (an additional 7.5%).<br>The intervention had a positive immediate effect on the willingness for HPV vaccination in parents, especially among fathers.<br>In the 3-month follow-up survey, 8.2% of parents responded that their daughters were vaccinated, with no difference in vaccination rates in the two groups.
Takahashi, Y et al. 2022 [49]	Randomized controlled trial	27 female university students who had received less than three doses of the HPV vaccine or were unvaccinated. Japan.	HPV	**Media**<br>**New technologies**<br>Intervention consists of medical information and educational tools on cervical cancer and HPV vaccination.<br>Participants were divided into three arms:<br>1. No intervention (control).<br>2. Distributed by mail.<br>3. Distributed through social networking service (LINE, Facebook, and Twitter) on websites.<br>All three arms are followed up for 15 months, during which, each intervention (Arms 2 and 3) is conducted three times at 6-month intervals.	Comparison of the survey between the control group and the intervention group in two rounds over a period of 15 months.<br>The questionnaires inquire about general information about the participant; history of HPV vaccination; knowledge about cervical cancer, using the health literacy scale; and assessment of the intervention by communicative and critical health literacy (CCHL) scale.	Participants' knowledge and health literacy improved post-intervention.<br>The results of the study are still preliminary, and comprehensive results are expected to be published after completing the study.
Davies, C et al. 2023 [35]	Randomized controlled trial	6957 adolescents aged 12-13 years from 40 schools. Australia.	HPV	**Community education**<br>**Media**<br>The adolescents were divided according to whether the school belonged to the control group or the intervention group.<br>Intervention schools delivered an intervention including three components:<br>1. In-class education and vaccination-day guidelines.<br>2. Decisional support tool booklet<br>3. Logistical component like comprised form return strategies, in-school catch-up of missed doses, etc.	Comparison between the control group and the intervention group about 3-dose HPV immunisation completion, the time to vaccinate students and proportion of consent form returns.	The intervention did not have a significant impact on the overall uptake of the HPV vaccine.<br>There was a small, non-significant difference in favour of the intervention group for each HPV vaccine dose: dose 1, 0.8%; dose 2, 0.2%; dose 3, 0.5%.

Author	Study Type	Sample size Population Country	Vaccine	Intervention	Evaluation	Conclusions
Froid-vaux, L et al. 2023 [50]	Quasi-experimental design.	14163 9th-grade students from the Canton of Vaud. Switzerland.	HPV	**Media**<br>Intervention group received a brochure containing information on the benefits and safety of HPV vaccination and address vaccine hesitancy. School nurses and physicians played a role in the intervention by informing students about vaccination, controlling immunization status, and offering scheduled administration of recommended vaccinations, including HPV.<br>Control group did not receive intervention.	Comparison of the vaccination rate among the intervention group (students of the 2019-2020 school year who did receive the new brochure) and the control group (Control Group 1 included students of the 2019-2020 school year who did not receive the new brochure; Control Group 2 included students of the 2017-2018 school year attending the same schools as the intervention group; Control Group 3 included students of the 2017-2018 school year who were not included in CG2).	The simple intervention of introducing new information material was insufficient to increase HPV vaccination coverage among the targeted students.<br>Multivariate analyses revealed that the 2019-2020 school year and being 13 years of age or older were significantly associated with vaccine uptake.
Liu, R et al. 2023 [36]	Randomized controlled trial	518 patients diagnosed with heart failure from 11 hospitals in Henan Province. China.	Influenza	**Community education**<br>The intervention will include three components:<br>1. Education to all members of the health care team, physicians, nurses, and patients:<br>– A 30-minute training session to health care teams in all participating hospitals.<br>– A 15-minute group or individual training session to patients. The sessions include content on association between influenza infection and cardiovascular disease and recommendations of influenza vaccine for secondary prevention in patients with cardiovascular disease.<br>2. Provision of free influenza vaccine on the day of discharge.<br>3. Provision of immunization service inside hospital before discharge.<br>The control group continued with usual care, which involved advice at discharge to attend a community-based vaccination service.	Comparison of the influenza vaccine coverage, development of influenza-like illness (ILI) and the composite of death or HF-specific hospital readmission for heart failure between intervention and control hospitals during 3-months follow up.<br>Interviews were carried out with health professionals and patients to evaluate contextual factors, intervention, implementation fidelity, intervention's mechanism of impact, and outcome.	The intervention resulted in a high influenza vaccine coverage rate (VCR) of 89.9% in the intervention group, compared to only 0.6% in the control group.<br>The intervention was well-accepted and adopted by both patients and health professional and education successfully increased the awareness of the benefits for influenza vaccination among providers and patients.

Author	Study Type	Sample size Population Country	Vaccine	Intervention	Evaluation	Conclusions
Mourad, N et al. 2023 [51]	Quasi-experimental design.	2165 university students in the Bekaa Region. Lebanon.	Influenza	**Media**<br>**New technologies**<br>**In the 2017–2018 study**, the campaign "Beat the Flu" focused on influenza vaccination, during which information concerning influenza's signs and symptoms, methods of transmission, complications, and preventative measures was provided with thorough elaboration about vaccination. Educational material such as informative leaflets and fact sheets were handed out, and data regarding students' vaccination status were collected.<br>**In the 2021–2022 study**, a continuation of the "Beat the Flu" campaign was carried out online, using various social media platforms including Facebook and Instagram. The campaign material was sent to the students' emails to ensure its delivery to all, included the importance of influenza vaccination during the COVID-19 pandemic.	Comparison of the survey between 2015-2016, 2017-2018 and 2021-2022 studies.<br>The questionnaire was divided into two sections:<br>(1) demographic characteristics, and (2) vaccination status and reasons for non-vaccination.<br>The 2017–2018 and 2021–2022 studies included an addition of reasons for vaccination, whereas, in the 2021–2022 study, a section related to the effect of COVID-19 on influenza vaccination was added.	Most university students in the three studies did not receive the influenza vaccine, with refuse rates ranging from 84.7% to 89.2% across different influenza seasons despite the vaccination promotional programs and the COVID-19 pandemic.<br>The "Beat the Flu" campaign was only mentioned as a reason for acquiring the vaccine by between 25% and 11% of students for the 2017-2018 and 2021-2022 studies respectively.
Reno, J. E et al. 2023 [44]	Randomized controlled trial	700 Latinx young adults and parents of adolescents. USA.	HPV	**Tailored message**<br>**New technologies**<br>Intervention use the extended parallel processing model (EPPM) to design messages. The messages were divided into three different classes emphasizing the relative benefit of HPV vaccination in relation to (1) cancer prevention, (2) genital warts prevention, or (3) neither (control message that mentioned HPV without specific health risks).<br>The messages included a hyperlink to "more info," which directed participants to a note stating they would receive further information about HPV vaccination at the end of the survey.	Comparison of the pre- and post-intervention survey between the control group and the intervention group.<br>– In the pretest, HPV information seeking intention and participants' baseline likelihood to vaccinate for HPV were measured.<br>– In the post-test, HPV information seeking intention, vaccination intention, Message Fear Appeal, Perceived Severity and Susceptibility, Self-efficacy and Response efficacy of HPV vaccination and HPV information seeking.	Fear appeals, when combined with a link to more information about HPV vaccination, were effective in inspiring information seeking among those who had not yet decided to vaccinate against HPV.<br>Shorter messages with embedded hyperlinks were found to be more effective in inspiring information seeking about HPV vaccination compared to more complex messages.<br>The study did not find support for the hypothesis that parents would respond more acutely to cancer messages and young adults would respond more acutely to genital warts messages.

Author	Study Type	Sample size Population Country	Vaccine	Intervention	Evaluation	Conclusions
Reno, J. E et al. 2023 [45]	Randomized controlled trial	965 parents of adolescents ages 9-17 and young adults ages 18-26 in Denver. USA.	HPV	**Tailored message**<br>**New technologies**<br>3-armed trial that compared different strategies:<br>1. Individually tailored educational website intervention with messages to address the participant's most salient concerns about HPV vaccination (based on a pre-intervention survey): Combatting HPV Infection & Cancer (CHiCOS).<br>2. Untailored, web-based intervention adapted from the Centers for Disease Control and Prevention's "Vaccine Information Sheet" (VIS) for HPV.<br>3. "Usual care" (no intervention).	Comparison of the pre-, post-intervention and 3 months follow-up survey between the different arms.<br>The survey assess change in HPV vaccination intention, vaccination attitudes and values, intervention and website engagement and satisfaction, HPV vaccine decision quality and vaccination status.	Both the tailored and untailored web-based educational intervention conditions were effective at increasing decisional quality and intention to vaccinate for HPV.<br>Although there were no significant differences found between the tailored (CHiCOS) and untailored intervention conditions and neither the CHiCOS nor untailored condition saw significant increases in vaccine uptake compared to the usual care group; thus, increased intention to vaccinate did not result in vaccine behaviour change.
Songol, A et al. 2023 [37]	Randomized controlled trial	366 parents that delayed in vaccinating their children under 1 year of age with one or more doses of vaccine in Shooshtar. Iran.	General childhood vaccination	**Community education**<br>**Media**<br>The intervention provided education by dividing the population into two groups:<br>1. Group education session in the form of film-based with a duration of 13 min and 50 s based on Mayer's multimedia principles.<br>2. Lecture with the same educational content as the film group, for 15 to 20 min.<br>The program covered microbe-induced diseases, vaccine mechanisms, safety, and the importance of child vaccination., multi-vaccine administration, benefits, and side effects. It also tackled misconceptions, provided vaccination schedules, and aided forgetful parents.	Comparison of the post-intervention questionnaire between the different groups.<br>The questionnaire was designed in three parts:<br>1. Information about the parents.<br>2. Variables related to the child.<br>3. Questions about vaccination, including previous and current vaccinations, reasons for late injection, source of vaccine information, adherence to vaccination schedule, and response to opposition to vaccination. The questionnaire also included a question about the time interval between participating in the intervention and receiving the vaccine.	Education delivered via film was more effective in reducing the delay in vaccination compared to education delivered via lecture. The OR of delay in vaccination in the lecture group was 78 % higher than in the film group.<br>The chance of delay in vaccination after the intervention was higher in the lecture group than in the film group, with 51.8% of children in the lecture-based education group receiving their next vaccine with a delay of more than 7 days, compared to 37.6% in the film-based education group.

Table S6. Classification of articles according to the tool selected for assessing risk of bias

Study	*Tool*		*Study*	*Tool*
**Gagneur, A et al. 2018 [38]**	ROBINS-I		**Shegog, R et al. 2022 [58]**	RoB
**Costantino, C et al. 2019 [24]**	ROBINS-I		**Taddio, A et al. 2022 [32]**	RoB
**Freedman, T et al. 2019 [25]**	ROBINS-I		**Zhang, X et al. 2022 [33]**	RoB
**Kim, M et al. 2019 [52]**	RoB		**Salmon, D. A et al. 2019 [41]**	NIH quality assessment tool for before-after (Pre-Post) study without control group
**Blanchard, J-L et al. 2020 [26]**	NIH quality assessment tool for before-after (Pre-Post) study without control group		**Zhang, X et al. 2020 [34]**	ROBINS-I
**Glanz, J. M et al. 2020 [39]**	RoB		**Dudley, M et al. 2021 [42]**	RoB
**Icardi, G et al. 2020 [27]**	NIH quality assessment tool for before-after (Pre-Post) study without control group		**Ibraheem, R et al. 2021 [48]**	RoB
**Johri, M et al. 2020 [28]**	RoB		**Suzuki, Y et al. 2021 [59]**	RoB
**Nowak, G. J et al. 2020 [53]**	RoB		**Dudley, M. Z et al. 2022 [43]**	RoB
**Davies, C et al. 2021 [29]**	RoB		**Lubis, T. A et al. 2022 [60]**	ROBINS-I
**Di Mauro, A et al. 2021 [54]**	NIH quality assessment tool for before-after (Pre-Post) study without control group		**Suzuki, Y et al. 2022 [61]**	RoB
**Lecce, M et al. 2021 [55]**	NIH quality assessment tool for before-after (Pre-Post) study without control group		**Takahashi, Y et al. 2022 [49]**	RoB
**Suryadevara, M et al. 2021 [46]**	ROBINS-I		**Davies, C et al. 2023 [35]**	RoB
**Yousuf, H et al. 2021 [56]**	RoB		**Froidevaux, L et al. 2023 [50]**	ROBINS-I
**Bethke, N et al. 2022 [30]**	RoB		**Liu, R et al. 2023 [36]**	RoB
**Boccalini, S et al. 2022 [31]**	NIH quality assessment tool for before-after (Pre-Post) study without control group		**Mourad, N et al. 2023 [51]**	ROBINS-I
**Keselman, A et al. 2022 [47]**	RoB		**Reno, J. E et al. 2023 [44]**	RoB
**Lewin, E. B et al. 2022 [57]**	RoB		**Reno, J. E et al. 2023 [45]**	RoB
**Muñoz -Miralles, R et al. 2022 [40]**	RoB		**Songol, A et al. 2023 [37]**	RoB

Table S7. Studies assessed with NIH quality assessment tool for before-after (Pre-Post) study without control group

Questions	Study					
	Blanchard, J-L et al. 2020 [26]	*Icardi, G et al. 2020 [27]*	*Di Mauro, A et al. 2021 [54]*	*Lecce, M et al. 2021 [55]*	*Boccalini, S et al. 2022 [31]*	*Salmon, D. A et al. 2019 [41]*
**1. Was the study question or objective clearly stated?**	Yes	Yes	Yes	Yes	Yes	No
**2. Were eligibility/selection criteria for the study population pre-specified and clearly described?**	No	Yes	Yes	No	Yes	Yes
**3. Were the participants in the study representative of those who would be eligible for the test/service/intervention in the general or clinical population of interest?**	CD	NR	No	CD	No	Yes
**4. Were all eligible participants that met the prespecified entry criteria enrolled?**	No	CD	Yes	No	No	CD
**5. Was the sample size sufficiently large to provide confidence in the findings?**	Yes	CD	NR	Yes	Yes	Yes
**6. Was the test/service/intervention clearly described and delivered consistently across the study population?**	Yes	Yes	Yes	Yes	Yes	Yes
**7. Were the outcome measures prespecified, clearly defined, valid, reliable, and assessed consistently across all study participants?**	Yes	Yes	Yes	Yes	Yes	No
**8. Were the people assessing the outcomes blinded to the participants' exposures/interventions?**	No	NR	NR	NR	No	NR
**9. Was the loss to follow-up after baseline 20% or less? Were those lost to follow-up accounted for in the analysis?**	Yes	Yes	Yes	NR	Yes	NA
**10. Did the statistical methods examine changes in outcome measures from before to after the intervention? Were statistical tests done that provided p values for the pre-to-post changes?**	Yes	Yes	Yes	Yes	Yes	Yes
**11. Were outcome measures of interest taken multiple times before the intervention and multiple times after the intervention (i.e., did they use an interrupted time-series design)?**	No	No	No	No	No	No
**12. If the intervention was conducted at a group level (e.g., a whole hospital, a community, etc.) did the statistical analysis take into account the use of individual-level data to determine effects at the group level?**	Yes	Yes	No	Yes	Yes	NA

* CD, cannot determine; NA, not applicable; NR, not reported

Table S8. Studies assessed with ROBINS-I tool for non-randomized studies of interventions

**Study**	**Questions**							**ROBINS-I overall bias**
	**Domain 1: Cofounding**	**Domain 2: Selection**	**Domain 3: Classification of interventions**	**Domain 4: Deviation from interventions**	**Domain 5: Missing data**	**Domain 6: Measurement of outcomes**	**Domain 7: Selection of reported result**	
**Gagneur, A et al. 2018 [38]**	Low	Low	Low	Low	Low	Low	Moderate	Moderate
**Costantino, C et al. 2019 [24]**	Moderate	Low	Low	Low	Low	Low	Low	Moderate

Study	Questions							
	Domain 1: Cofounding	Domain 2: Selection	Domain 3: Classification of interventions	Domain 4: Deviation from interventions	Domain 5: Missing data	Domain 6: Measurement of outcomes	Domain 7: Selection of reported result	ROBINS-I overall bias
Freedman, T et al. 2019 [25]	Low	Low	Low	Low	Low	Moderate	Moderate	Moderate
Suryadevara, M et al. 2021 [46]	Serious	Low	Low	Low	Low	Moderate	Low	Serious
Zhang, X et al. 2020 [34]	Low	Low	Low	Low	Low	Moderate	Low	Moderate
Lubis, T. A et al. 2022 [60]	Low	Low	Low	Low	Low	Moderate	Low	Moderate
Froidevaux, L et al. 2023 [50]	Moderate	Low	Low	Low	Serious	Moderate	Low	Serious
Mourad, N et al. 2023 [51]	Moderate	Low	Low	Low	Low	Moderate	Low	Moderate

Table S9. Studies assessed with RoB 2 tool for in randomized trials

	Questions							
	Domain 1a: Randomization process	Domain 1b: identification or recruitment of participants[t]	Domain S: Period and carryover effects[tt]	Domain 2: Deviations from intended interventions	Domain 3: Missing outcome data	Domain 4: Measurement of the outcome	Domain 5: Selection of the reported result	RoB overall bias
Kim, M et al. 2019 [52]	Low	NA	NA	Low	Low	Some concerns	Some concerns	Some concerns
Glanz, J. M et al. 2020 [39]	Low	NA	NA	Low	Low	Low	Low	Low
Johri, M et al. 2020 [28]	Low	Low	NA	Some concerns	Low	Low	Low	Some concerns
Nowak, G. J et al. 2020 [53]	Low	NA	NA	Some concerns	Low	Some concerns	Low	Some concerns
Davies, C et al. 2021 [29]	Low	Low	NA	Some concerns	Low	Low	Low	Some concerns
Yousuf, H et al. 2021 [56]	Low	NA	NA	Low	Low	Low	Low	Low
Bethke, N et al. 2022 [30]	Some concerns	High	NA	Low	Some concerns	Some concerns	High	High
Keselman, A et al. 2022 [47]	High	NA	NA	Some concerns	Low	Some concerns	Low	High
Lewin, E. B et al. 2022 [57]	Low	NA	NA	Low	Low	Low	Low	Low
Muñoz -Miralles, R et al. 2022 [40]	Low	Low	NA	Low	Low	Low	Low	Low

	Questions							RoB overall bias
	**Domain 1a: Randomization process**	**Domain 1b: identification or recruitment of participants[t]**	**Domain S: Period and carryover effects[tt]**	**Domain 2: Deviations from intended interventions**	**Domain 3: Missing outcome data**	**Domain 4: Measurement of the outcome**	**Domain 5: Selection of the reported result**	
**Shegog,R et al. 2022 [58]**	Low	Low	NA	Low	Low	Low	Low	Low
**Taddio, A et al. 2022 [32]**	Low	Low	NA	Low	Low	Low	Low	Low
**Zhang, X et al. 2022 [33]**	Low	Low	NA	Low	Low	Low	Low	Low
**Dudley, M et al. 2021 [42]**	Low	Low	NA	Low	Some concerns	Low	Low	Some concerns
**Ibraheem, R et al. 2021 [48]**	Low	NA	NA	Low	Low	Low	Low	Low
**Suzuki, Y et al. 2021 [59]**	Low	NA	NA	Low	Low	Low	Low	Low
**Dudley, M. Z et al. 2022 [43]**	Low	NA	NA	Low	Low	Low	Low	Low
**Suzuki, Y et al. 2022 [61]**	Low	NA	NA	Low	Low	Low	Low	Low
**Takahashi, Y et al. 2022 [49]**	Low	Low	NA	Some concerns	Low	Low	Low	Some concerns
**Davies, C et al. 2023 [35]**	High	Low	NA	Some concerns	Low	Some concerns	Low	High
**Liu, R et al. 2023 [36]**	Low	Low	NA	Low	Low	Low	Low	Low
**Reno, J. E et al. 2023 [44]**	Low	NA	NA	Low	Low	Low	Low	Low
**Reno, J. E et al. 2023 [45]**	Low	NA	NA	Low	Low	Low	Low	Low
**Songol, A et al. 2023 [37]**	Low	Low	NA	Low	Low	Low	Low	Low

* NA, not assessable; t, only available for cluster-randomized trials; tt, only available for cross-over trials.
